# Next-generation ensemble projections reveal higher climate risks for marine ecosystems

**DOI:** 10.1038/s41558-021-01173-9

**Published:** 2021-10-21

**Authors:** Derek P. Tittensor, Camilla Novaglio, Cheryl S. Harrison, Ryan F. Heneghan, Nicolas Barrier, Daniele Bianchi, Laurent Bopp, Andrea Bryndum-Buchholz, Gregory L. Britten, Matthias Büchner, William W. L. Cheung, Villy Christensen, Marta Coll, John P. Dunne, Tyler D. Eddy, Jason D. Everett, Jose A. Fernandes-Salvador, Elizabeth A. Fulton, Eric D. Galbraith, Didier Gascuel, Jerome Guiet, Jasmin G. John, Jason S. Link, Heike K. Lotze, Olivier Maury, Kelly Ortega-Cisneros, Juliano Palacios-Abrantes, Colleen M. Petrik, Hubert du Pontavice, Jonathan Rault, Anthony J. Richardson, Lynne Shannon, Yunne-Jai Shin, Jeroen Steenbeek, Charles A. Stock, Julia L. Blanchard

**Affiliations:** 1 Department of Biology, Dalhousie University, Halifax, Nova Scotia Canada; 2 United Nations Environment Programme World Conservation Monitoring Centre, Cambridge, UK; 3 Institute for Marine and Antarctic Studies, University of Tasmania, Hobart, Tasmania Australia; 4 Center for Marine Socio-ecology, University of Tasmania, Hobart, Tasmania Australia; 5 School of Earth, Environmental and Marine Science, University of Texas Rio Grande Valley, Port Isabel, TX USA; 6 Department of Ocean and Coastal Science and Centre for Computation and Technology, Louisiana State University, Baton Rouge, LA USA; 7 School of Mathematical Sciences, Queensland University of Technology, Brisbane, Queensland Australia; 8 MARBEC, IRD, Univ Montpellier, Ifremer, CNRS, Sète/Montpellier, France; 9 Department of Atmospheric and Oceanic Sciences, University of California Los Angeles, Los Angeles, CA USA; 10 LMD/IPSL, CNRS, Ecole Normale Supérieure, Université PSL, Sorbonne Université, Ecole Polytechnique, Paris, France; 11 Program in Atmospheres, Oceans, and Climate, Massachusetts Institute of Technology, Cambridge, MA USA; 12 Potsdam-Institute for Climate Impact Research (PIK), Potsdam, Germany; 13 Institute for the Oceans and Fisheries, The University of British Columbia, Vancouver, British Columbia Canada; 14 Institute of Marine Science (ICM-CSIC), Barcelona, Spain; 15 Ecopath International Initiative Research Association, Barcelona, Spain; 16 NOAA/OAR Geophysical Fluid Dynamics Laboratory, Princeton, NJ USA; 17 Centre for Fisheries Ecosystems Research, Fisheries and Marine Institute, Memorial University of Newfoundland, St. John’s, Newfoundland and Labrador Canada; 18 School of Mathematics and Physics, The University of Queensland, St. Lucia, Queensland Australia; 19 Commonwealth Scientific and Industrial Research Organisation (CSIRO) Oceans and Atmosphere, Queensland Biosciences Precinct, St Lucia, Brisbane, Queensland Australia; 20 Centre for Marine Science and Innovation, The University of New South Wales, Sydney, New South Wales Australia; 21 AZTI, Marine Research, Basque Research and Technology Alliance (BRTA), Sukarrieta (Bizkaia), Spain; 22 Commonwealth Scientific and Industrial Research Organisation (CSIRO) Oceans and Atmosphere, Hobart, Tasmania Australia; 23 Department of Earth and Planetary Science, McGill University, Montreal, Quebec Canada; 24 UMR Ecology and Ecosystems Health (ESE), Institut Agro, Inrae, Rennes, France; 25 NOAA Fisheries, Woods Hole, MA USA; 26 Department of Biological Sciences, University of Cape Town, Cape Town, South Africa; 27 Center for Limnology, University of Wisconsin, Madison, WI USA; 28 Department of Oceanography, Texas A&M University, College Station, TX USA; 29 Atmospheric and Oceanic Sciences Program, Princeton University, Princeton, NJ USA

**Keywords:** Marine biology, Ecological modelling, Climate-change ecology

## Abstract

Projections of climate change impacts on marine ecosystems have revealed long-term declines in global marine animal biomass and unevenly distributed impacts on fisheries. Here we apply an enhanced suite of global marine ecosystem models from the Fisheries and Marine Ecosystem Model Intercomparison Project (Fish-MIP), forced by new-generation Earth system model outputs from Phase 6 of the Coupled Model Intercomparison Project (CMIP6), to provide insights into how projected climate change will affect future ocean ecosystems. Compared with the previous generation CMIP5-forced Fish-MIP ensemble, the new ensemble ecosystem simulations show a greater decline in mean global ocean animal biomass under both strong-mitigation and high-emissions scenarios due to elevated warming, despite greater uncertainty in net primary production in the high-emissions scenario. Regional shifts in the direction of biomass changes highlight the continued and urgent need to reduce uncertainty in the projected responses of marine ecosystems to climate change to help support adaptation planning.

## Abstract (web summary)

Use of an enhanced suite of marine ecosystem models and Earth system model outputs from Phase 6 of the Coupled Model Intercomparison Project (CMIP6) reveals greater decline in mean global ocean animal biomass than previously projected under both strong-mitigation and high-emissions scenarios.

## Main

Anthropogenic climate change is a growing threat to marine ecosystems^1^, with impacts projected to intensify a suite of organismal responses, including increased mortality, reduced calcification and changes to species distributions, interactions, abundance and biomass^2,3^. Furthermore, climate change can interact with other stressors such as overfishing^4,5^, which can threaten marine conservation^6^ and societal benefits derived from the ocean^7,8^. Thus, understanding the risks of climate change for marine ecosystems and the benefits of mitigation is paramount.

Projecting the magnitude and impacts of climate change through model intercomparison projects (MIPs) produces ensemble projections that quantify inter-model spread (the range of projections from low to high)^9^ and stimulate long-term efforts to develop and improve models. The most prominent MIP, the Coupled Model Intercomparison Project (CMIP)^10^, is currently on its sixth phase of Earth system model (ESM) simulation experiments, forming a core contribution to the sixth Intergovernmental Panel on Climate Change Assessment Report (IPCC AR6). These simulation outputs can also be used to drive impact models to explore how climate change will affect specific human sectors or natural processes.

While individual marine ecosystem models (MEMs) have explored climate impacts on ocean ecosystems, the Fisheries and Marine Ecosystem Model Intercomparison Project (Fish-MIP) compares models produced by different modelling groups into standardized ensemble projections^11^. Fish-MIP has explored a range of topics, including global^12^ and regional^13–15^ changes over the coming century and their potential socioeconomic consequences^8^.

All Fish-MIP contributions to date have been driven by CMIP5 ESM outputs. However, the ‘next-generation’ CMIP6 ESMs provide an updated suite of oceanographic drivers^16,17^. In this article, we compare MEM ensembles forced with CMIP5 and CMIP6 variants of the Geophysical Fluid Dynamics Laboratory (GFDL) and the Institut Pierre-Simon Laplace (IPSL) ESMs. CMIP6’s next-generation ESMs capture improved representations of marine biogeochemistry^18^, sea ice and other oceanographic properties, and the GFDL simulation has a higher spatial resolution. The bias of CMIP6 models was reduced by 20–70% for surface nitrate, phosphate and silicate relative to CMIP5, and the root mean squared error for surface temperature and chlorophyll improved^17,18^. CMIP6 models exhibit increased climate sensitivity (the equilibrium response of mean surface air temperature to a doubling of atmospheric CO_2_) over CMIP5^19^, resulting in generally stronger marine ecosystem forcings (for example, ocean warming) under the high-emissions scenario, although with more variation in terms of impacts on net primary productivity (NPP)^17,20^.

Three global MEMs have been added to the Fish-MIP ensemble, bringing the total to nine (Table 1). In this article, we present results from the newly expanded suite of MEMs under CMIP6 high-emissions and strong-mitigation scenarios. We focus on temperature and productivity as key drivers of marine ecosystem change as these variables are used by all MEMs (Supplementary Table 1). We also provide a direct comparison between the subset of MEMs that used both CMIP5 and CMIP6 forcings to evaluate the consequences of improved next-generation climate models for global marine ecosystem projections and to assess mitigation benefits. The revised Fish-MIP simulation protocol is kept as similar as possible to the CMIP5 protocol to enable direct comparison, including using the same two ESMs (albeit from different generations) and focusing on climate impacts on total unfished marine animal biomass.

**Table 1:** List of global MEMs participating in the study and differences relative to CMIP5 analysis

MEM	Model type^11^	CMIP5	CMIP6	Key forcing variables used	Summarized differences between CMIP5 and CMIP6 MEM structure (if any)	Taxonomic groups included	Key reference(s)
APECOSM	Composite (size- and trait-based; functional group structure)	x	x	Carbon concentrations (small phytoplankton, large phytoplankton, small zooplankton, large zooplankton), particulate organic matter (small and large), zonal and meridional currents, turbulent mixing, temperature, water density, dissolved oxygen concentration, light irradiance. All fields 3D and monthly.	To avoid problems with the 3D interpolation of forcings, APECOSM was run on the native ORCA1 grid using the native IPSL-CM set of forcing fields. The 3D outputs were then vertically integrated and interpolated on the 2D Fish-MIP 1° x 1° grid. Minor improvements include fine tuning of some parameters and bug fixes with minor impacts on the outputs.	Epipelagic fish, migratory mesopelagic fish, resident mesopelagic fish	^39,40^
BOATS	Size-based	x	x	Mean temperature 0–75 m, NPP	None	All commercially fished species, both finfish and invertebrates	^41,42^
DBEM	Species distribution model	x	x	Surface and bottom O_2_, pH, salinity and temperature. Ice cover, current velocity, NPP, NPP pico and NPP diat. All variables on a yearly basis.	None	956 species of exploited fishes and invertebrates	^43,44^
DBPM	Composite (size- and trait-based)	x	x	Surface and bottom temperature, phytoplankton carbon groups	None	All benthic and pelagic marine animals weighing between 1 mg and 1 tonne	^45^
EcoOcean	Composite (trophodynamic and species distribution model)	x	x	SST, seafloor temperature, column average temperature, phytoplankton carbon groups	(1) Improved representation of species contributions to ecosystem dynamics, (2) improved responses of the marine food web to stratified environmental drivers	Includes 51 functional groups representing the whole spectrum of marine organisms from bacteria to whales, and integrates explicit information for 3,400 species of vertebrates, invertebrates and primary producers	^29,46^
EcoTroph	Trophic-level-based		x	NPP, SST, integrated mesozooplankton carbon	None	Implicitly all groups, including pelagic and demersal fishes and invertebrates	^37,47^
FEISTY	Composite		x	Seafloor temperature, seafloor detritus flux, mean temperature 0–100 m, integrated mesozooplankton carbon 0–100 m	None	Small pelagic fish, large pelagic fish, demersal fish, benthic invertebrates	^48^
Macroecological	Size-based	x	x	NPP, SST	None	Implicitly all marine organisms from 1 gram to 1 tonne	^49^
ZooMSS	Composite (size- and trait-based; functional group structure)		x	Chlorophyll-a, SST	None	Flagellates, cilliates, omnivorous copepods, carnivorous copepods, larvaceans, salps, chaetognaths, euphausiids, jellyfish, fish	^50^

All models produce monthly outputs on a 1° × 1° grid (except DBEM, which produces yearly outputs on a 0.5° × 0.5° grid). 2D, two-dimensional; 3D, three-dimensional.

## ESM projections

**Fig. 1:**
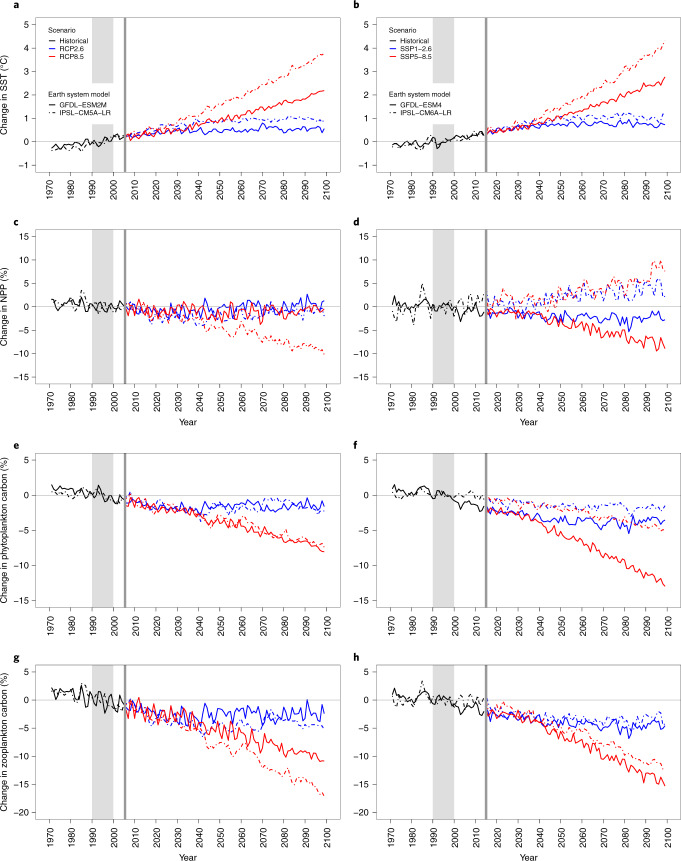
Projected mean global change in oceanographic properties from IPSL and GFDL ESMs. **a**–**h**, Rows depict SST (**a**,**b**), NPP (**c**,**d**), phytoplankton carbon (**e**,**f**) and zooplankton carbon (**g**,**h**) for GFDL and IPSL CMIP5 and CMIP6 under strong-mitigation (blue) and high-emissions (red) scenarios. Historical values (1970–2005 for CMIP5; 1970–2014 for CMIP6) are shown in black, and projections (2006–2099 for CMIP5; 2015–2099 for CMIP6) are coloured. All values are normalized relative to the period 1990–1999. Vertical grey shaded area indicates reference decade, and vertical grey line indicates first year of projection (subsequent to historical period). RCP, representative concentration pathway.

**Extended Data Fig. 1:**
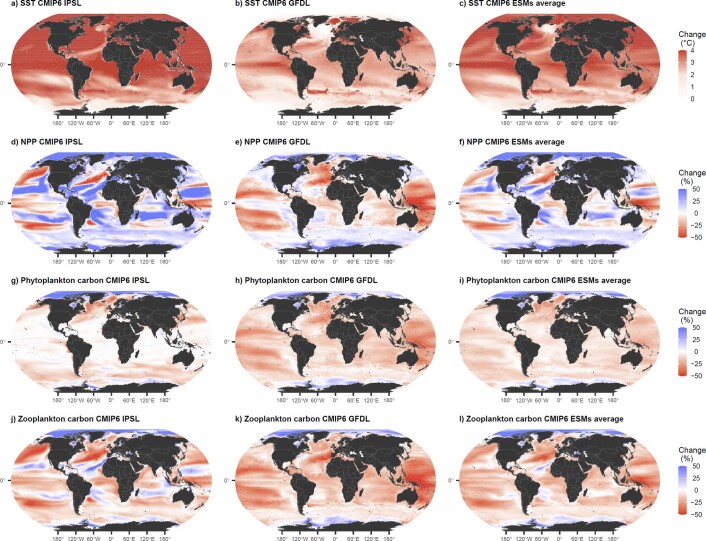
Spatial change in oceanographic properties under CMIP6 SSP5-8.5. Change in sea -surface temperature (**a**-**c**), net primary production (**d**-**f**), phytoplankton carbon (**g**-**i**) and zooplankton carbon (**j**-**l**) between 1990–1999 and 2090–2099. Left column shows the mean change for IPSL-CM6A-LR. Middle column shows the mean change for GFDL-ESM4. Rightmost column shows the mean change averaged across GFDL and IPSL.

The IPSL and GFDL CMIP6 ESM simulations show a stronger mean surface warming of the global ocean from the 1990s to the 2090s relative to the CMIP5 ESM simulations in the high-emissions scenario but a reduced difference in the strong-mitigation scenario (Fig. 1a,b). By contrast, there is more diversity in projected changes in NPP (Fig. 1c,d) and plankton biomass (Fig. 1e-h). Under CMIP5, only IPSL under high emissions shows an overall decline in global NPP, of ~10% by 2090–2099 (Fig. 1c). All other simulations (GFDL for both scenarios and IPSL for strong mitigation) exhibit little overall global change in NPP. However, under both CMIP6 scenarios, GFDL shows up to 5–10% decline in global NPP, whereas IPSL projects an increase of the same magnitude, with stronger responses under high emissions in both cases (Fig. 1d). The NPP increase in IPSL CMIP6 is especially prominent in subtropical gyres (Extended Data Fig. 1), and preliminary analysis suggests this could be linked to a warming-induced increase in di-nitrogen fixation^17^. Importantly, despite increasing NPP, the phytoplankton and zooplankton biomasses in IPSL show marked declines for CMIP6, as was the case in CMIP5, although with greater differences between the two ESMs (Fig. 1e-h).

**Fig. 2:**
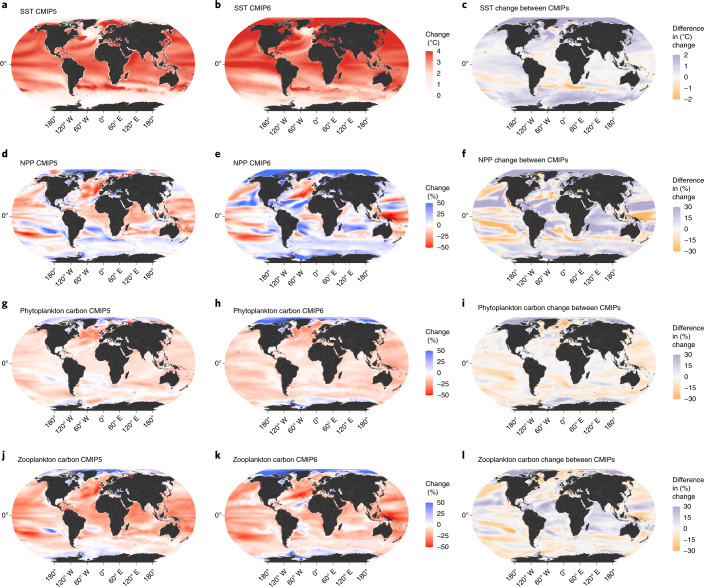
Projected mean spatial changes in oceanographic properties. **a**–**l**, Rows depict SST (**a**–**c**), NPP (**d-f**), phytoplankton carbon (**g**–**i**) and zooplankton carbon (**j**–**l**) for 1990–1999 and 2090–2099. Maps represent mean change for GFDL and IPSL models under a high-emissions scenario. Columns depict mean change under CMIP5 (**a**,**d**,**g**,**j**), mean change under CMIP6 (**b**,**e**,**h**,**k**) and the difference in these century changes between CMIP6 and CMIP5 (**c**,**f**,**i**,**l**), with a positive value indicating a stronger increase (or weaker decrease) in CMIP6, a negative value indicating a weaker increase (or stronger decrease) in CMIP6 and zero representing an equal change projected in both CMIP5 and CMIP6.

**Extended Data Fig. 2:**
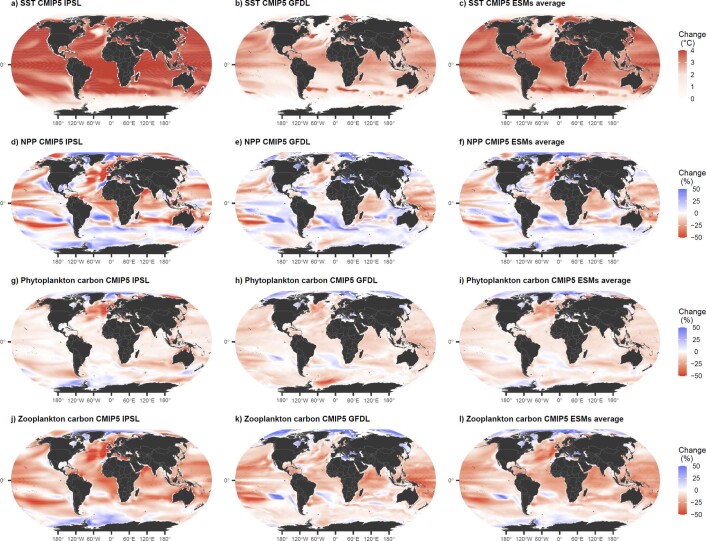
Spatial change in oceanographic properties under CMIP5 RCP8.5. Change in sea surface temperature (**a**-**c**), net primary production (**d**-**f**), phytoplankton carbon (g-**i**) and zooplankton carbon (**j**-**l**) between 1990–1999 and 2090–2099. Left column shows the mean change for IPSL-CM5A-LR. Middle column shows the mean change for GFDL-ESM2M. Rightmost column shows the mean change averaged across GFDL and IPSL.

Spatially, IPSL and GFDL CMIP6 ESMs project sea surface temperature (SST) increases across almost the entire global ocean under the high-emissions scenario (Fig. 2b), with the largest changes in the Arctic, northern temperate regions (except the centre of the North Atlantic subpolar gyre) and a belt around the Equator. Polar oceans show a generally broader surface warming in CMIP6 simulations than in CMIP5 (Fig. 2c and Extended Data Figs. 1 and 2) as well as the highest NPP increases over the twenty-first century under the high-emissions scenario (Fig. 2e and Extended Data Figs. 1 and 2). The Pacific and North Atlantic oceans contain large areas of projected NPP decrease (Fig. 2e), although of reduced extent relative to CMIP5 (Fig. 2d,e). The Arctic shows a blanket increase under CMIP6, seemingly driven by IPSL differences relative to CMIP5 (Extended Data Figs. 1 and 2). Changes in phytoplankton and zooplankton biomass are more consistently negative, decreasing everywhere except the poles (Fig. 2g-l), and more regionally variable for zooplankton (Fig. 2j–l). The spatial congruence between CMIP5 and CMIP6 in the direction of changes for plankton biomasses, however, was greater than for NPP (Fig. 2e,h,k).

## MEM projections

**Fig. 3:**
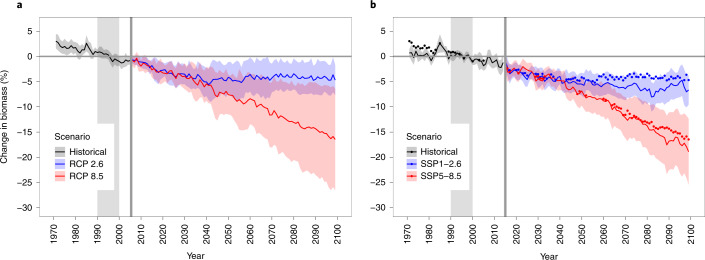
Multimodel mean change in marine animal biomass under strong-mitigation and high-emissions scenarios. **a**, CMIP5. **b**, CMIP6. Blue colouring represents strong mitigation, and red represents high emissions. Coloured dots indicate years with higher ensemble means for CMIP5. All values relative to standardized reference period (1990–1999). Solid coloured lines indicate ensemble means; shaded areas indicate inter-model standard deviation. Vertical grey shaded area and line indicate reference decade and first year of projection after historical period, respectively. The full ensemble of MEMs is included for CMIP5 (7 models using IPSL and 5 models using GFDL, *n* = 12) and CMIP6 (9 models using IPSL and 7 models using GFDL, *n* = 16).

**Fig. 4:**
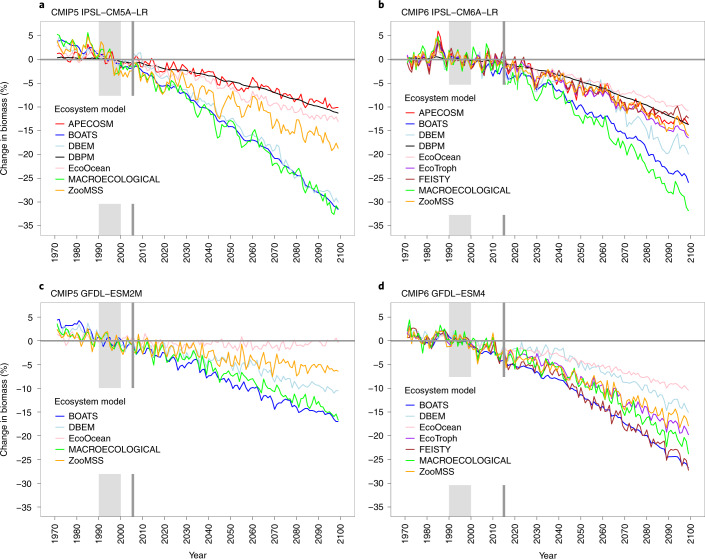
Projected global change in marine animal biomass from individual MEMs driven by IPSL and GFDL under the high-emissions scenario. **a**, CMIP5 IPSL-CM5A-LR. **b**, CMIP6 IPSL-CM6A–LR. **c**, CMIP5 GFDL-ESM2M. **d**, CMIP6 GFDL-ESM4M. A different set of MEMs is included for CMIP5 (7 models using IPSL and 5 models using GFDL, *n* = 12) and CMIP6 (9 models using IPSL and 7 models using GFDL, *n* = 16). All values are relative to the standardized reference period of 1990–1999. Vertical grey shaded area indicates reference decade, and vertical grey line indicates first year of projection subsequent to historical period.

**Extended Data Fig. 3:**
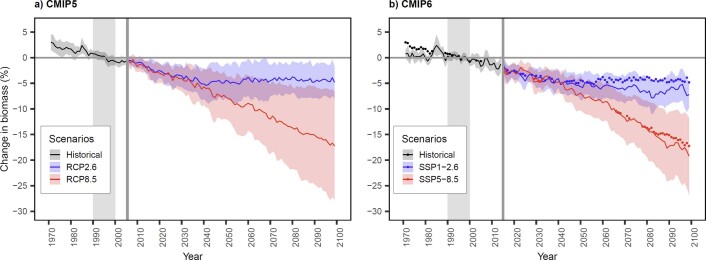
Multi-model change in marine animal biomass across comparable set of six marine ecosystem models (MEMs) run using both CMIP5 and CMIP6 forcings. CMIP5 (**a**) and CMIP6 (**b**) results averaged across GFDL and IPSL under strong-mitigation (blue) and high-emissions (red) scenarios. Solid lines indicate ensemble model means; shaded areas indicate + /- inter-model standard deviation (n = 10; two MEMs only used IPSL). All values are relative to the standardized reference period of 1990–1999 (vertical grey shaded area). Vertical grey shaded area indicates reference decade and vertical grey line indicates first year of projection (subsequent to historical period). For the suite of MEMs considered, see Fig. 4. Coloured dots indicate CMIP5 values in years in which the ensemble mean values were higher for CMIP5 than for CMIP6.

**Extended Data Fig. 4:**
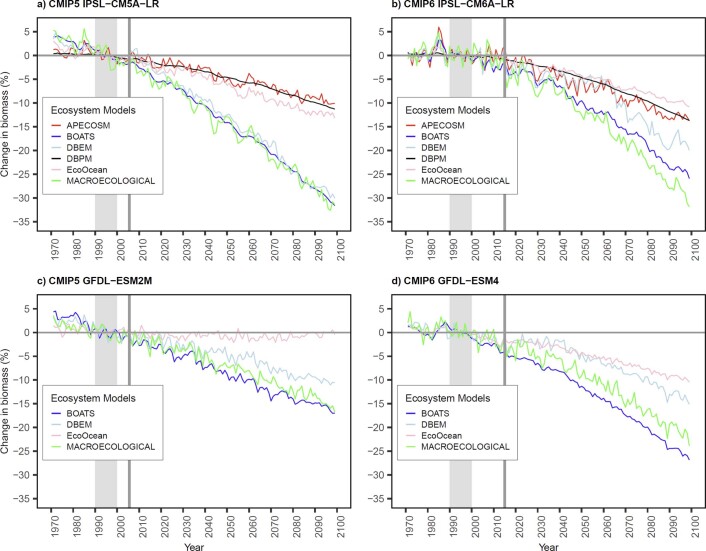
Projected global change in marine animal biomass across comparable set of six marine ecosystem models (MEMs) run using both CMIP5 and CMIP6 forcings under the high-emissions scenario. MEM outputs using CMIP5 (a & c) and CMIP6 (b & b) forcings (n = 10; two MEMs only used IPSL). All values are relative to the standardized reference period of 1990–1999. Vertical grey shaded area indicates reference decade and vertical grey line indicates first year of projection (subsequent to historical period).

Mean projected global marine animal biomass from the full MEM ensemble shows no clear difference between the CMIP5 and CMIP6 simulations until ~2030 (Fig. 3). After 2030, CMIP6-forced models show larger declines in animal biomass, with almost every year showing a more pronounced decrease under strong mitigation and most years from 2060 onwards showing a more pronounced decrease under high emissions (Fig. 3). Both scenarios have a significantly stronger decrease in 2090–2099 under CMIP6 than CMIP5 (two-sided Wilcoxon rank-sum test on annual values; *n* = 160 for CMIP6, 120 for CMIP5; *W* = 12,290 and *P* < 0.01 for strong mitigation, *W* = 11,221 and *P* = 0.016 for high emissions). For the comparable MEM ensemble (Extended Data Fig. 3), only the strong-mitigation scenario is significantly different (*n* = 120 for both CMIPs; *W* = 6,623 and *P* < 0.01). The multiple consecutive decades in which CMIP6 projections are more negative than CMIP5 (Fig. 3b and Extended Data Fig. 3b) suggest that these results are not due simply to decadal variability in the selected ESM ensemble members. Under high emissions, the mean marine animal biomass for the full MEM ensemble declines by ~19% for CMIP6 by 2099 relative to 1990–1999 (~2.5% more than CMIP5), and the mitigation scenario declines by ~7% (~2% more than CMIP5). Similar declines were observed for the comparable MEM ensemble. Notably, the ensemble inter-model standard deviations show a total separation between high-emissions and strong-mitigation scenarios under CMIP6 after the 2080s for both the full and comparable MEM ensembles, whereas they overlap under CMIP5 (Fig. 3b and Extended Data Fig. 3). This was due primarily to a narrowing of the inter-model standard deviation for the high-emissions scenario; all the CMIP6 GFDL-forced MEMs project a higher rate of biomass decrease in the latter half of the twenty-first century (Fig. 4c,d and Extended Data Fig. 4c,d), which brings those models’ projections closer to those of IPSL-forced models.

**Fig. 5:**
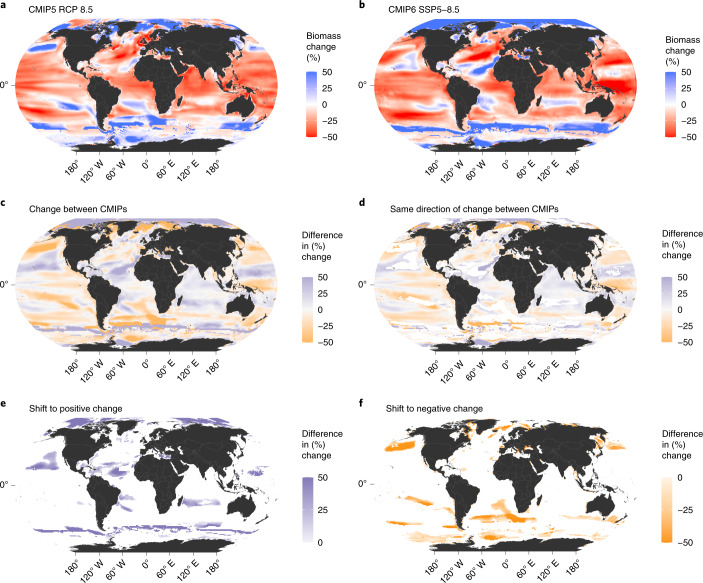
Ensemble mean change in marine animal biomass under the high-emissions scenario. The full ensemble of MEMs is included for CMIP5 (7 models using IPSL and 5 models using GFDL, *n* = 12) and CMIP6 (9 models using IPSL and 7 models using GFDL, *n* = 16). **a**,**b**, Maps represent mean percentage change between 1990–1999 and 2090–2999 under CMIP5 (**a**) and CMIP6 (**b**). **c**, Difference in percentage change between CMIP5 and CMIP6. **d**, Difference in percentage change between CMIP5 and CMIP6 for grid cells showing same direction of change. **e**, Difference in percentage change between CMIP5 and CMIP6 for grid cells changing from CMIP5 decrease to CMIP6 increase. **f**, Same as in **e** for grid cells changing from CMIP5 increase to CMIP6 decrease.

**Extended Data Fig. 5:**
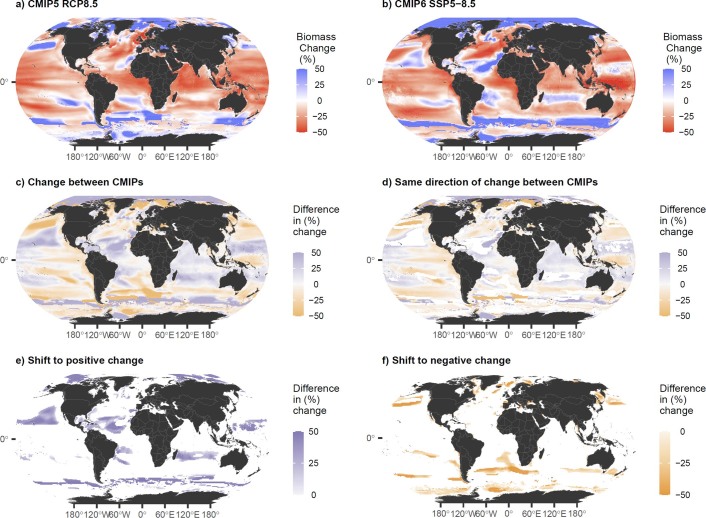
Ensemble mean change in marine animal biomass across comparable set of six marine ecosystem models (MEMs) that used both CMIP5 and CMIP6 forcings under the high-emissions scenario. MEM outputs using CMIP5 (**a**) and CMIP6 (**b**) forcings from GFDL and IPSL (n = 10; two MEMs only used IPSL). Maps represent mean percentage change between 1990–1999 and 2090–2999 under a) CMIP5 and b) CMIP6; **c**) difference in percentage change between CMIPs; **d**) difference in percentage change between CMIPs for grid cells that showed the same direction of change; **e**) difference in percentage change between CMIPs for grid cells that changed from a decrease in CMIP5 to an increase in CMIP6 and **f**) vice versa.

**Extended Data Fig. 6:**
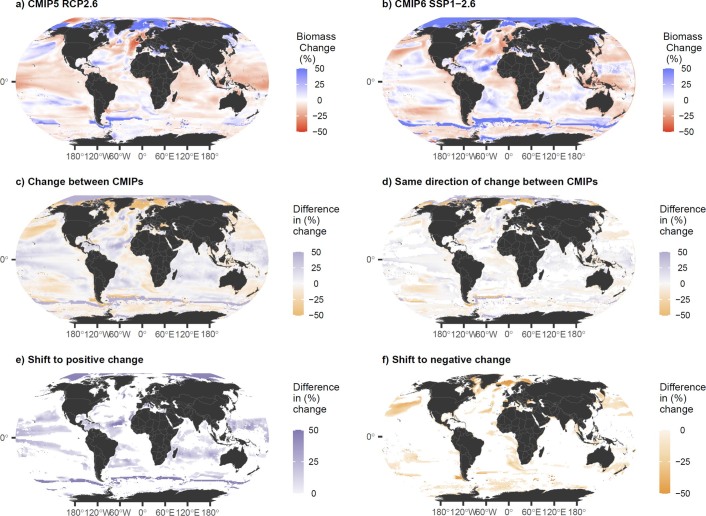
Ensemble mean change in marine animal biomass across comparable set of six marine ecosystem models (MEMs) that used both CMIP5 and CMIP6 forcings under the high-mitigation scenario. MEM outputs using CMIP5 (**a**) and CMIP6 (**b**) forcings from GFDL and IPSL (n = 10; two MEMs only used IPSL). Maps represent mean percentage change between 1990–1999 and 2090–2999 under a) CMIP5 and b) CMIP6; **c**) difference in percentage change between CMIPs; **d**) difference in percentage change between CMIPs for grid cells that showed the same direction of change; **e**) difference in percentage change between CMIPs for grid cells that changed from a decrease in CMIP5 to an increase in CMIP6 and **f**) vice versa.

Biomass changes across the full and comparable MEM ensembles show similar global spatial patterns between CMIP5 and CMIP6 under both scenarios (Fig. 5a,b and Extended Data Figs. 5a,b and 6a,b). However, there are substantial regional differences (Fig. 5a–c and Extended Data Figs. 5a–c and 6a–c). In all cases, CMIP6 MEM ensemble means project an increase in animal biomass essentially everywhere in the Arctic by the 2090s, whereas CMIP5 projects both increases and decreases in the region. The pattern is spatially heterogeneous for other regions, although equatorial regions do show a consistent biomass decline for both CMIP5 and CMIP6 (Fig. 5a–c and Extended Data Figs. 5a–c and 6a–c). In total, 71% of grid cells indicate the same direction of change for the ensemble model biomass under CMIP5 and CMIP6, with 15% switching from decreases to increases (Fig. 5d) and another 14% vice versa (Fig. 5e). Among the areas that change, large parts of the Arctic Ocean show positive changes, and a large area of temperate latitudes exhibits negative changes (Fig. 5d–e).

**Extended Data Fig. 7:**
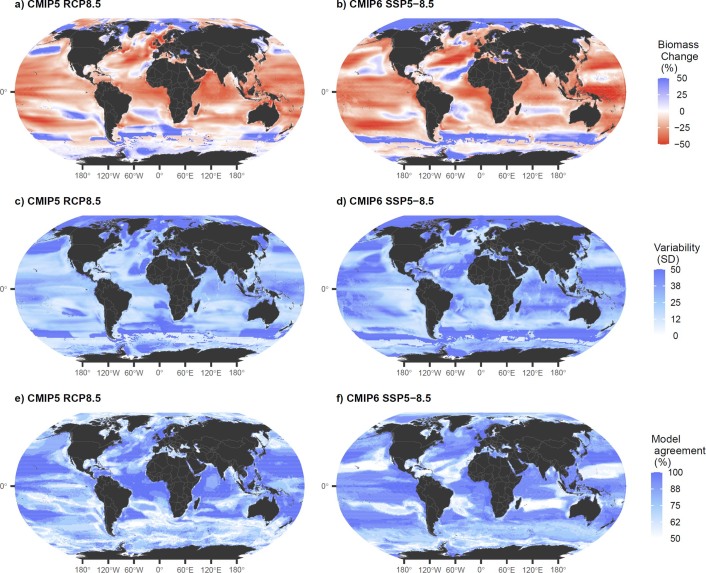
Ensemble model results from the full set of global marine ecosystem models under the high-emissions scenario. Total consumer biomass change (**a**, **b**), inter-model standard deviation (**c**, **d**), and model agreement (**e**, **f**) where 100% represents all models indicating the same direction of change, and 50% indicates half the models indicating one direction of change and half indicating the opposite.

**Extended Data Fig. 8:**
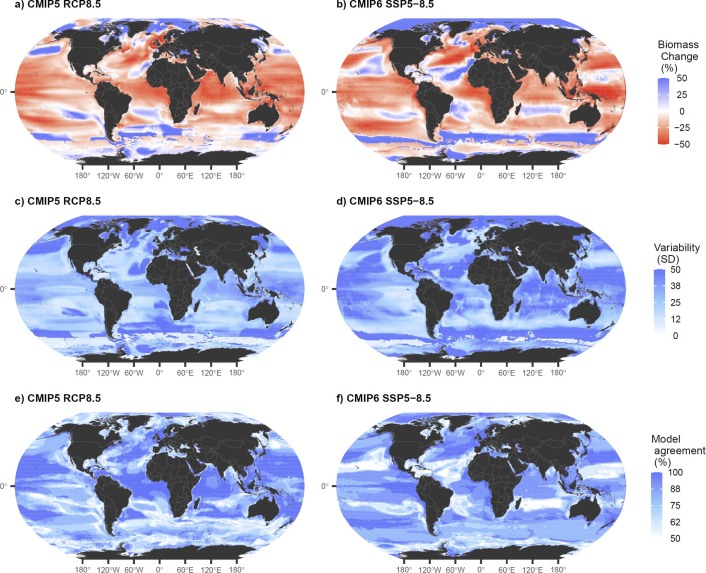
Ensemble model results from the comparable set of global marine ecosystem models that ran both CMIP5 and CMIP6 simulations under the high-emissions scenario. Total consumer biomass change (**a**, **b**), inter-model standard deviation (**c**, **d**), and model agreement (**e**, **f**) where 100% represents all models indicating the same direction of change, and 50% indicates half the models indicating one direction of change and half indicating the opposite.

**Extended Data Fig. 9:**
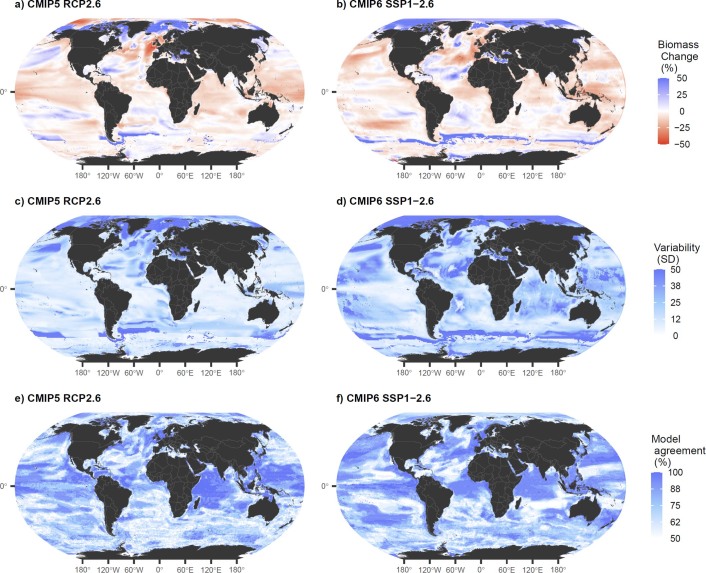
Ensemble model results from the full set of global marine ecosystem models under the strong-mitigation scenario. Total consumer biomass change (**a**, **b**), inter-model standard deviation (**c**, **d**), and model agreement (**e**, **f**) where 100% represents all models indicating the same direction of change, and 50% indicates half the models indicating one direction of change and half indicating the opposite.

**Extended Data Fig. 10:**
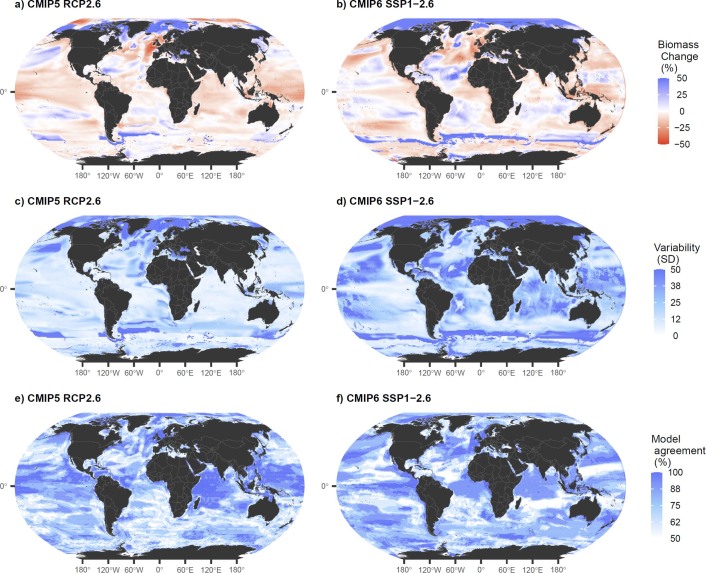
Ensemble model results from the comparable set of global marine ecosystem models that ran both CMIP5 and CMIP6 simulations under the strong-mitigation scenario. Total consumer biomass change (**a**, **b**), inter-model standard deviation (**c**, **d**), and model agreement (**e**, **f**) where 100% represents all models indicating the same direction of change, and 50% indicates half the models indicating one direction of change and half indicating the opposite.

Notably, the MEM model agreement (Extended Data Fig. 7e,f) is fairly high (>80%) in most oceanic regions under CMIP6, except for the subtropical gyres where the agreement drops to 50% (maximum disagreement among models), probably due to the marked differences in CMIP6 NPP and the various ways MEMs incorporate lower-trophic-level forcing^21^. Regardless of whether the full or comparable MEM models are considered (Extended Data Figs. 8, 9 and 10), there is no clear spatial improvement in model agreement compared with CMIP5.

## Discussion and conclusions

Our comparison of GFDL and IPSL climate drivers for MEM projections revealed a substantial spatial reshuffling of projected marine animal biomass change in the global ocean between CMIP5 and CMIP6. Overall, these changes suggest that when averaged across the ensemble, total marine animal biomass will decline more steeply when forced by CMIP6 ESMs than by CMIP5 (Fig. 3), with a greater separation between high-emissions and strong-mitigation scenarios emphasizing the benefits of mitigation (Fig. 3 and Extended Data Fig. 3). Differences in projected biomass changes appeared to be caused primarily by increased climate sensitivity of CMIP6 simulations^22^, specifically the IPSL and GFDL simulations used here. This is supported by the comparable MEM ensemble results (Extended Data Figs. 3–5 and 8).

Warming can affect metabolic costs, rates of biomass production, mortality and species distributions and interactions in individual MEMs; a detailed study of the response of individual MEMs to warming in isolation^21^ revealed a substantial variability in mechanisms and responses, although a broadly consistent negative impact on biomass. Warming is also accompanied by increased ocean stratification and a marked biomass decrease of non-nitrate-fixing phytoplankton^23^. Combining these mechanisms, the pattern is one of consistent global animal biomass decline under both CMIP5 and CMIP6, with a strengthened decline under CMIP6.

The impact of changing productivity and biomass in the lower trophic levels modelled in ESMs remains complex. Biogeochemical forcing variables, particularly NPP, were substantially altered under the high-emissions scenario in CMIP6, with directional differences between the two ESMs at the global scale (Fig. 1d), although the phytoplankton and zooplankton biomasses that support upper trophic levels decrease in a very similar way to CMIP5. Four of nine MEMs use NPP as their primary input (BOATS, DBEM, EcoTroph and MACROECOLOGICAL), while others are forced by phytoplankton and/or zooplankton biomass or a proxy thereof (DBPM, EcoOcean and ZooMSS) or combine plankton biomass with particulate organic matter (APECOSM and FEISTY) to generate new animal production (Table 1 and Supplementary Table 1). These differences within Fish-MIP represent structural variability in the MEMs, and the sensitivity of the results, as well as the general agreement, should be seen as a test of how robust the result of declining MEM biomass under climate change is to the ecological and other assumptions of the MEMs. Variability in how lower trophic levels are included in MEMs can result in a range of directional changes even under the same climate simulation experiment, highlighting the need for improvement in the coupling of MEMs with biogeochemical variable outputs^21^. However, the fact that all MEMs projected declining biomass under high emissions despite these differences around lower-trophic-level forcing suggests a robustness to the results and perhaps the particular importance of temperature effects^21^.

While the ESMs exhibited generally consistent trends in NPP and export in response to warming^24^, disagreement in the regional patterns of these responses and their global signature is an ongoing issue^25^. The inclusion of strong temperature-dependent remineralization, for example, can enhance recycling and NPP despite increasing stratification under ocean surface warming, yet this is treated unevenly across ESMs. Variations in light harvesting and nutrient uptake parameters within broad uncertainty bounds can shift latitudinal thresholds between nutrient- and light-limited regimes in biogeochemical models, with the latter likely to be positively impacted by increasing stratification under climate change and the former negatively^26^. Regional circulation changes, which often vary strongly between models, can shape regional NPP trends^27^. Efforts to constrain factors controlling the NPP response to climate change^28^ or leverage emergent constraints to reduce NPP projection uncertainty^23^ are ongoing but did not result in convergence for CMIP6. We also caution that none of the MEMs (or ESMs for plankton) yet includes the potential for adaptation or evolution of individual taxa and that there remains the lack of a full bi-directional coupling between higher- and lower-trophic-level (biogeochemical) processes in the MEMs. Furthermore, many species interactions are not well captured by global-scale MEMs, and the potential for nonlinear tipping points that cause rapid ecological deterioration remains unclear^21^.

Projected mean changes in animal biomass had a smaller spread across CMIP6-forced Fish-MIP models under both scenarios (Fig. 3). However, combining opposing NPP signals from ESMs that have also effectively become more similar in terms of warming response could create an illusion of model improvement and reduced uncertainty, although, as described, not all MEMs used NPP. The reduced spread of MEM projections is evident only when ESMs are averaged; within each ESM the spread of MEM biomass projections is similar under CMIP5 and CMIP6 (Fig. 4). Individual improvements in two MEMs between CMIP5 and CMIP6 could also have impacted results. For example, EcoOcean has recently been updated by improving its representation of specific species contributions to ecosystem dynamics and the response of the marine food web to different environmental drivers^29^. However, the majority of MEMs were unchanged (Table 1), so this is likely to have had a limited impact relative to the changes in ESM forcings.

While here we explore uncertainties in future animal biomass through generating an ensemble of impact models (MEMs) forced by standardized inputs from two contrasting ESMs and low and high carbon emission scenarios, we recognize that our treatment of uncertainty associated with internal climate variability is limited. However, this is generally a smaller source of uncertainty at the multi-decadal to century time scales explored here than model and scenario uncertainty for most fisheries-relevant biogeochemical drivers^30,31^. Nonetheless, future efforts to fully characterize these uncertainties associated with internal climate variability will be important for integrating an additional source of uncertainty into the ensembles, as will the addition of further ESMs, although here our experimental design aimed to use the same two ESMs as in the CMIP5 experiment^11,12^.

Sensitivity to climate responses in MEMs could also be examined across a range of past modelled and observed biogeochemical and/or ecological variables that might constrain responses to climate change, so-called emergent constraints^23^, as a means to improve both suites of ensembles. In addition, the greater integration of other changing biogeochemical components (such as oxygen) to more MEMs (Supplementary Table 1) would enable an ensemble exploration of a greater range of climate impacts. With increasing maturity of MEMs and communication between climate simulation and impact communities, such as through the CMIP6 Vulnerability, Impacts, Adaptation and Climate Services Advisory Board^16^, these opportunities should arise in the future.

In addition, and importantly, the MEM outputs explored here focus on total animal biomass without the inclusion of fishing impacts, which can act synergistically with climate change^4^. Consequently, projected changes in an exploited ocean may be larger. While restoring overfished stocks and limiting exploitation to sustainable levels may help with climate change adaptation at the regional and global levels^32^, our CMIP6 results suggest that there are larger challenges ahead for future fisheries potential than previously anticipated. Fisheries production potential has remained essentially flat for the past 40 years despite an increase in fishing effort, with marine capture fisheries landings on the order 0.1 Gt yr^–1^ (ref. ^33^). If CMIP6 projections hold, wild-capture fishery contributions to global food security may be further challenged.

As per previous Fish-MIP studies^8,12^, our results focus on total (potential) ecosystem biomass, rather than the ‘edible’ biomass available for fisheries, in part because we are interested in the overall ecosystem response to environmental change. In addition, given the heterogeneous nature of the MEMs, only a proportion of which are species-based, total ecosystem biomass is a variable that is consistently comparable across models. The ensemble outputs that we provide here complement organismal^34^ and spatial^35^ studies of climate impacts and vulnerabilities, both in terms of using such studies to further inform the development of individual MEMs and to provide ecosystem-level projections of trends and uncertainties due to changes in both temperature and productivity that provide additional context to the more finely resolved studies of thermal niches and life-history responses. For robust projections of edible fish biomass, we would also need scenarios of future fleet behaviour, economics and changes in target fishery species that are not yet available (and not included in Shared Socioeconomic Pathways (SSPs)). Projected decreases of global animal biomass do not necessarily imply that global fisheries catches would be reduced in proportion; changes in animal production, where explored in individual MEMs, suggest a similar climate response to biomass, although lower in magnitude^36,37^. We recognize that the inclusion of fishing impacts, which can act synergistically with climate change^4^, remains an important concern, and Fish-MIP is in the process of developing scenarios to enable such comparisons.

Our CMIP6 projections of twenty-first-century climate change impacts show steeper global biomass declines and thus greater climate risks for marine ecosystems than their CMIP5 counterparts forced by the same two ESMs, and emphasize the benefits of strong mitigation. Marked shifts in directional differences for many regions of the global ocean, probably driven by differences in ESM forcing, and in particular NPP, highlight the large uncertainties that still exist, suggesting that the readiness of ESM-forced global-scale MEMs to support country-level adaptation policies is still nascent, although these capabilities may be more advanced for regional models^38^. There remains an urgent need for model refinement to tackle uncertainty at all levels, including both climate and marine ecosystem projections. Only once these uncertainties have been addressed, so that climate-to-ecosystem modelling is improved, will the projections of climate impacts on marine organisms and fisheries be more robust and thus more strategically useful. A more ambitious model evaluation of the whole ocean including ecological-to-human coupled systems is required to deliver the rigorous projections urgently needed to advance climate adaptation and mitigation.

## Methods

The full ensemble approach used for Fish-MIP model intercomparisons, MEM architectures and the selection of ESM simulation outputs used as forcing variables is previously described^11^, but briefly, monthly ocean physical and biogeochemical outputs from ESMs run under prescribed scenarios are interpolated in space and time to a regular monthly 1° grid. These variables are then used under a common simulation protocol to force individual MEMs, with each modelling group using all relevant variables for their model (Supplementary Table 1). MEM outputs are then archived in a standardized 1° grid format for a common range of ecosystem variables.

For the Fish-MIP simulation round here, to enable the best possible comparison between CMIP5 and CMIP6, we utilized the same two ESMs: GFDL-ESM2M^51,52^ and IPSL-CM5A-LR^53^ for CMIP5 and the equivalent new generations for CMIP6, GFDL-ESM4.1^54–56^ and IPSL-CM6A-LR^57–59^. These were originally chosen on the basis of key criteria, including that they spanned a substantial fraction of the range of CMIP5 ESM projections in relevant oceanographic variables^11^. We compared projections under the strong-mitigation RCP 2.6 (CMIP6 SSP1–2.6) and the high-emissions RCP 8.5 (CMIP6 SSP5–8.5) scenarios. For the CMIP5-forced Fish-MIP model runs, the historical simulations spanned 1970–2005, and the RCP scenarios spanned 2006–2099; for CMIP6, the historical simulations spanned 1970–2014, and the SSP scenarios spanned 2015–2099. To enable a standardized comparison, a historical baseline period of 1990–1999 was used, with changes in MEM outputs evaluated relative to this period. To further enable direct comparison and isolation of impacts, climate change was the only stressor imposed on marine ecosystems, without fishing or other anthropogenic pressures superimposed. The primary output variable examined was total global marine animal biomass, with the specific range of marine animals represented (for example, species or functional groups) varying among MEMs^11^ but generally including major taxonomic groups or major fish taxa (Table 1). Given this, we examined trajectories of relative animal biomass change for each model over the twenty-first century rather than absolute values. For details on model calibration and validation, see ref. ^12^; for key MEM reference papers, see Table 1. The complete updated Fish-MIP protocol for CMIP6 forcings can be found at https://bit.ly/3jhWH7c.

In addition to the refined CMIP6 ESMs, two of the original six global Fish-MIP MEMs have also undergone improvements, reflecting the further development of parameterizations, additional processes and underlying hypotheses for the structure and dynamics of marine ecosystems (Table 1). Furthermore, three additional MEMs were added to the ensemble (EcoTroph^37,47^, FEISTY^48^ and ZooMSS^50^; Table 1). EcoTroph is a trophic-level-based model that implicitly includes all marine species of vertebrates and invertebrates; FEISTY is a composite model that includes both pelagic and demersal fish species as well as benthic invertebrates; ZooMSS is a composite model that includes fish but also focuses on resolving zooplankton taxa. In addition to examining projections across the entire expanded CMIP6 ensemble of nine MEMs (‘full MEM ensemble’), we also performed a more direct standardized comparison by using outputs from the CMIP5-driven ensemble of six MEMs against results from only these six MEMs for CMIP6 forcings (‘comparable MEM ensemble’). A large number of regional MEMs also contribute to Fish-MIP and warrant detailed investigation at the local scale, but they are not reported here for as straightforward comparison as possible.

### Reporting Summary

Further information on research design is available in the Nature Research Reporting Summary linked to this article.

## Online content

Any methods, additional references, Nature Research reporting summaries, source data, extended data, supplementary information, acknowledgements, peer review information; details of author contributions and competing interests; and statements of data and code availability are available at 10.1038/s41558-021-01173-9.

## Supplementary information

Supplementary Information Translated abstracts, Supplementary Figs. 1–10 and Table 1.

Reporting Summary

## Data Availability

All standardized forcing variables from the ESMs are available at 10.48364/ISIMIP.575744.1; all outputs from the MEMs are available via ISIMIP (https://www.isimip.org/gettingstarted/data-access/).

## Code availability

All code used to analyse simulations is available at https://github.com/Fish-MIP/CMIP5vsCMIP6.
