# Supplementary material for: Next-generation ensemble projections reveal higher climate risks for marine ecosystems
Source: Nat Clim Chang. 2021 Oct 21;11(11):973–81. doi: 10.1038/s41558-021-01173-9 (PMC8556156; doi:10.1038/s41558-021-01173-9)
Supplement: Supplementary file 1 — Translated abstracts, Supplementary Figs. 1–10 and Table 1. [file 41558_2021_1173_MOESM1_ESM.pdf]

nature portfolio

https://doi.org/10.1038/s41558-021-01173-9

Supplementary information

Next-generation ensemble projections reveal higher climate risks for marine ecosystems

In the format provided by the authors and unedited

Supplementary Information

Translated abstracts

Resumen (Español)

Las proyecciones de los impactos del cambio climático en los ecosistemas marinos han revelado una disminución a largo plazo en la biomasa global de animales marinos e impactos desiguales en las pesquerías mundiales. En el presente estudio se combina un conjunto mejorado de modelos de ecosistemas marinos globales del Proyecto de Intercomparación de Modelos de Ecosistemas Marinos y Pesqueros (Fish-MIP), con los modelos de nueva generación del sistema terrestre de la Fase 6 del Proyecto de Intercomparación de Modelos Acoplados (CMIP6), para realizar diversas proyecciones sobre cómo el cambio climático afectará los ecosistemas marinos futuros. En comparación con la combinación de la generación anterior de Fish-MIP-CMIP5, el nuevo conjunto de simulaciones de ecosistemas marinos muestra una mayor disminución en el promedio global de biomasa animal marina tanto en escenarios de fuerte mitigación como de altas emisiones debido al calentamiento elevado. En este último escenario, esto sucede a pesar de una mayor incertidumbre en la producción primaria neta. Los cambios regionales de dirección de los impactos a la biomasa destacan una necesidad continua y urgente de reducir la incertidumbre en las proyecciones de los ecosistemas marinos al cambio climático.

Resumo (Português)

As projeções dos impactos da mudança climática sobre os ecossistemas marinhos têm revelado declínios de longo prazo na biomassa animal marinha global e impactos desigualmente distribuídos na pesca. No presente estudo, se combina um conjunto aprimorado de modelos do ecossistema marinho global do Projeto de Intercomparação do Modelo de Pesca e do Ecossistema Marinho (Fish-MIP), com modelos da nova geração do sistema terrestre da Fase 6 do Projeto de Intercomparação do Modelo Acoplado (CMIP6), para explorar como a mudança climática afetará os ecossistemas marinhos futuros. Em comparação com o conjunto da geração anterior Fish-MIP-CMIP5, as novas simulações do ecossistema marinho global mostram um declínio maior na biomassa animal média do oceano em cenários de forte mitigação e alta emissão devido ao aquecimento elevado, apesar da maior incerteza na produção primária neta no cenário de altas emissões. Câmbios regionais na direção das mudanças de biomassa destacam a necessidade contínua e urgente de reduzir a incerteza nas respostas projetadas dos ecossistemas marinhos à mudança climática.

A) Expanded results from main manuscript

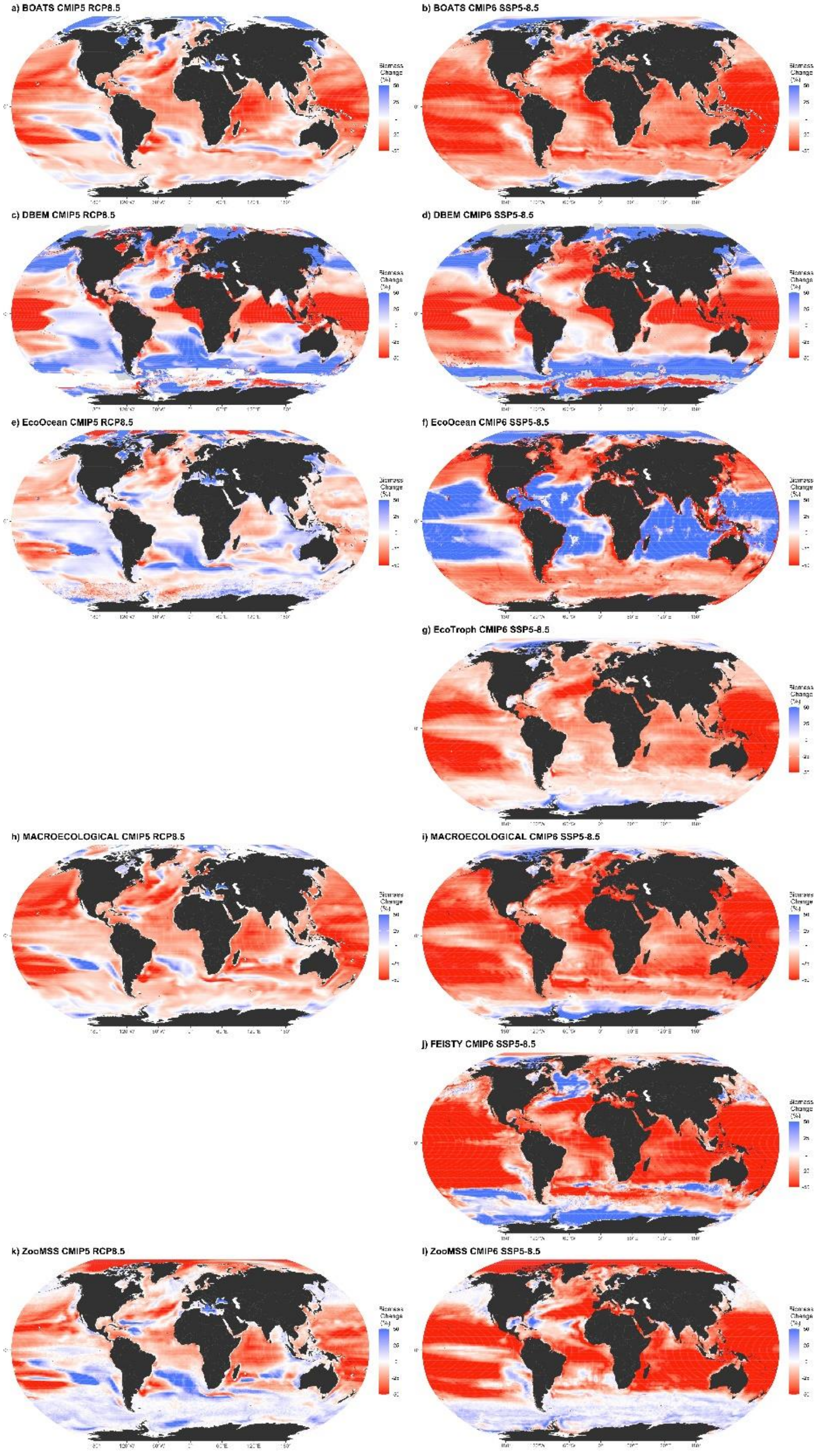

Supplementary Figure 1: Change in marine animal biomass between 1990-1999 and 2090-2099 for marine ecosystem models forced by GFDL CMIP5 (left column) and CMIP6 (right column) outputs under the high-emissions scenarios.

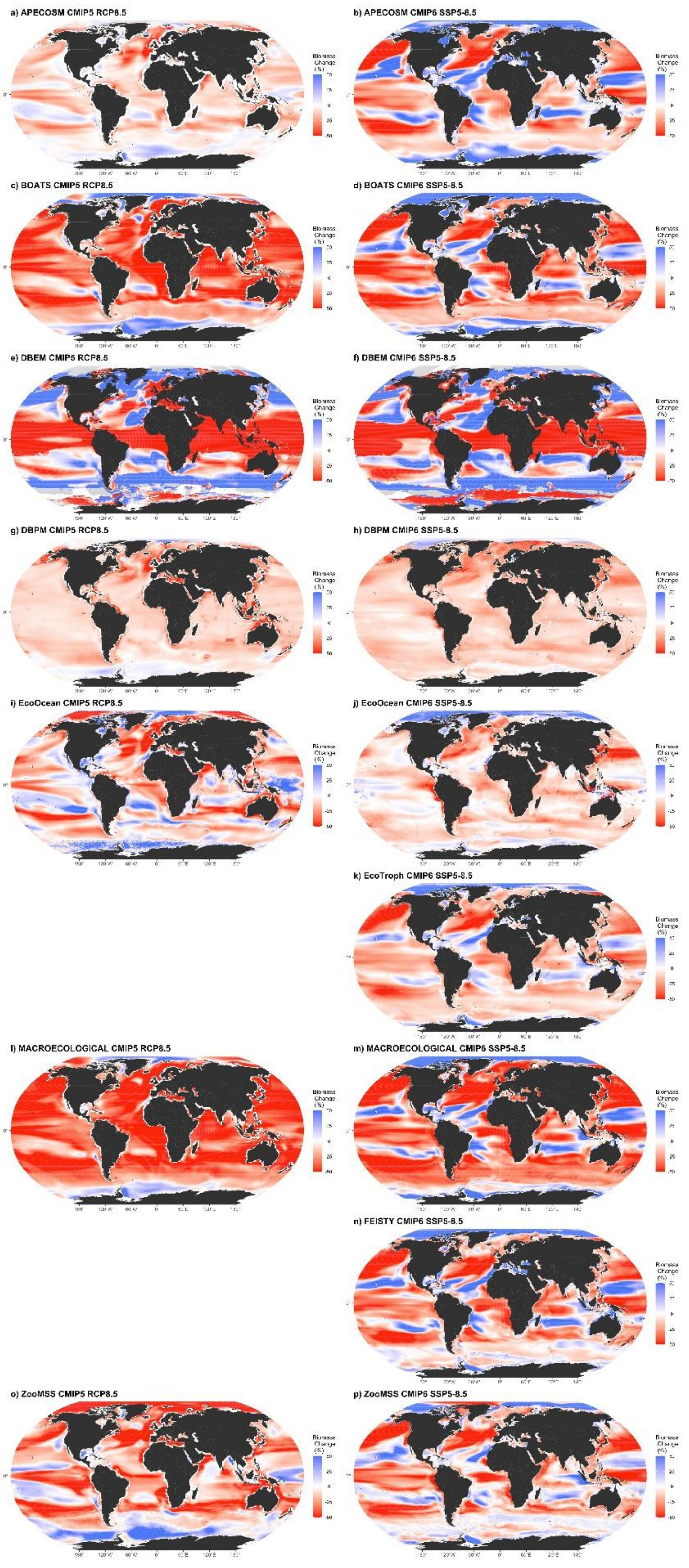
a) APECOSM CMIP5 RCP8.5
b) APECOSM CMIP6 SSP5-8.5
c) BOATS CMIP5 RCP8.5
d) BOATS CMIP6 SSP5-8.5
e) DBEM CMIP5 RCP8.5
f) DBEM CMIP6 SSP5-8.5
g) DBPM CMIP5 RCP8.5
h) DBPM CMIP6 SSP5-8.5
i) EcoOcean CMIP5 RCP8.5
j) EcoOcean CMIP6 SSP5-8.5
k) EcoTroph CMIP6 SSP5-8.5
l) MACROECOLOGICAL CMIP5 RCP8.5
m) MACROECOLOGICAL CMIP6 SSP5-8.5
n) FEISTY CMIP6 SSP5-8.5
o) ZooMSS CMIP5 RCP8.5
p) ZooMSS CMIP6 SSP5-8.5

Supplementary Figure 2: Change in marine animal biomass between 1990-1999 and 2090-2099 for each marine ecosystem model forced by IPSL CMIP5 (left column) and CMIP6 (right column) outputs under the high-emissions scenarios.

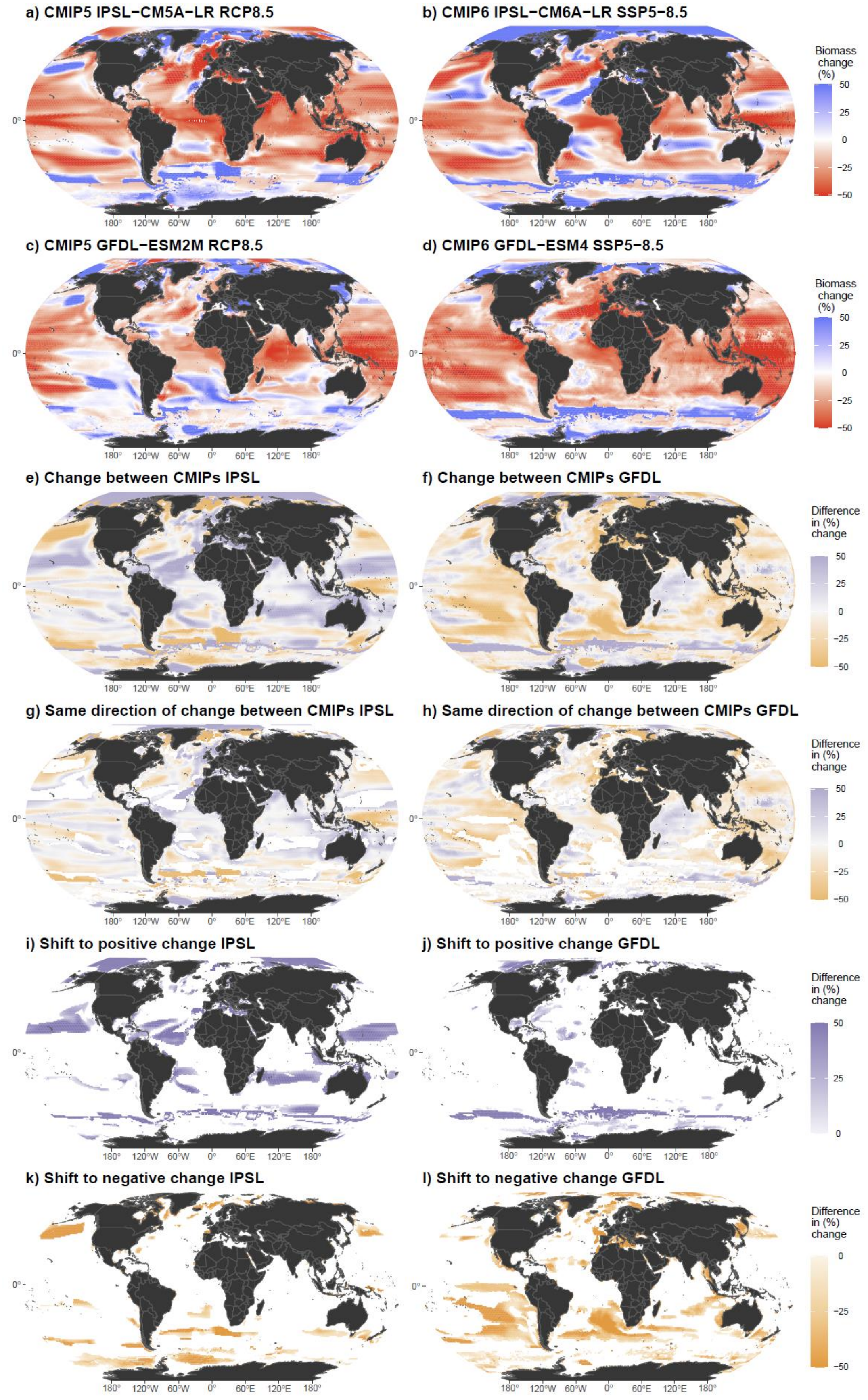
a) CMIP5 IPSL−CM5A−LR RCP8.5
b) CMIP6 IPSL−CM6A−LR SSP5−8.5
Biomass change (%)
50
25
0
−25
−50
c) CMIP5 GFDL−ESM2M RCP8.5
d) CMIP6 GFDL−ESM4 SSP5−8.5
Biomass change (%)
e) Change between CMIPs IPSL
f) Change between CMIPs GFDL
Difference in (%) change
g) Same direction of change between CMIPs IPSL
h) Same direction of change between CMIPs GFDL
Difference in (%) change
i) Shift to positive change IPSL
j) Shift to positive change GFDL
Difference in (%) change
k) Shift to negative change IPSL
l) Shift to negative change GFDL
Difference in (%) change
0°
180° 120°W 60°W 0° 60°E 120°E 180°

Supplementary Figure 3: Ensemble mean change in marine animal biomass across marine ecosystem models forced by GFDL and IPSL under the high- emissions scenario. Maps represent mean percent change between 1990-1999 and 2090-2999 under a) CMIP5 IPSL; b) CMIP6 IPSL; c) CMIP5 GFDL; and d) CMIP6 GFDL. Remaining maps represent (e-f) difference in percent change between CMIPs; (g-h) difference in percentage change between CMIPs for grid cells that showed the same direction of change; (i-j) difference in percent change between CMIPs for grid cells that changed from a decrease in CMIP5 to an increase in CMIP6; vice versa (k-l).

B) Additional results from full set of models under strong-mitigation scenario

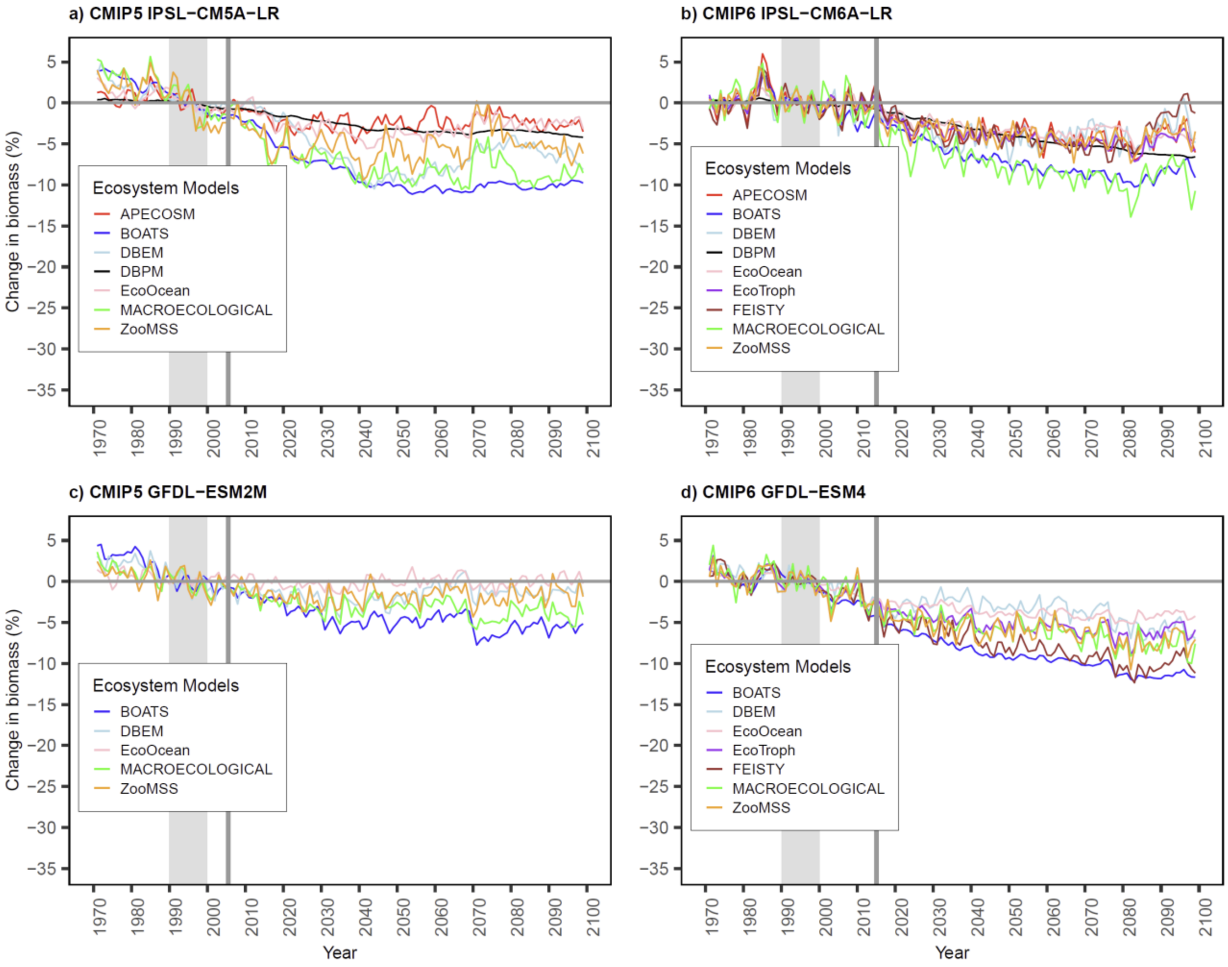

Supplementary Figure 4: Projected global change in marine animal biomass from individual marine ecosystem models (MEMs) using CMIP5 forcings from IPSL and GFDL under the strong-mitigation scenario (a and c) and CMIP6 forcings (b and d). A different set of MEMs is included for CMIP5 (7 models using IPSL forcings and 5 models using GFDL forcings, n = 12) and CMIP6 (9 models using IPSL forcings and 7 models using GFDL forcings, n = 16). All values are relative to the standardized reference period of 1990-1999. Vertical grey shaded area indicates reference decade and vertical grey line indicates first year of projection (subsequent to historical period).

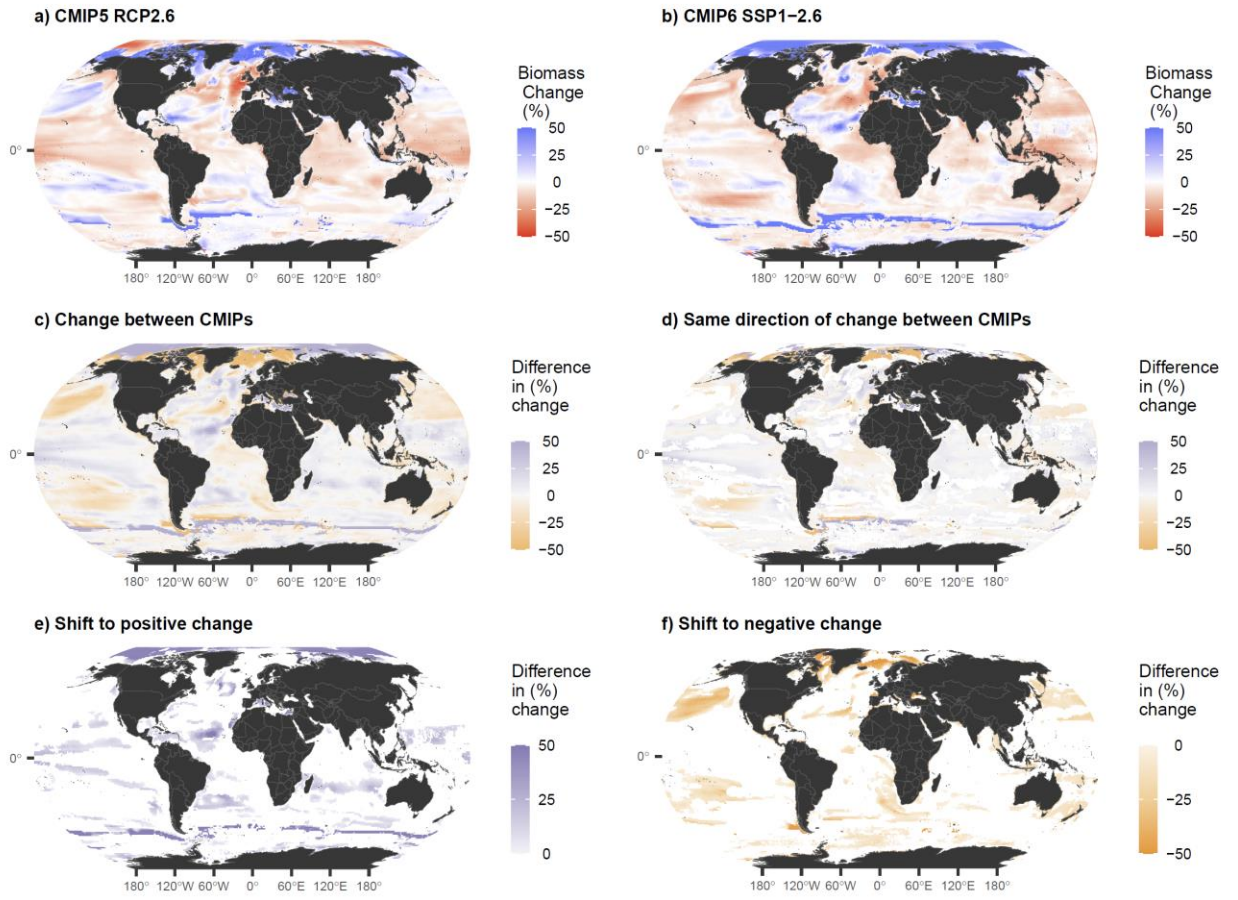

Supplementary Figure 5: Ensemble mean change in marine animal biomass across marine ecosystem models (MEMs) including those that only ran CMIP6 simulations. Forcings from GFDL and IPSL under the strong-mitigation scenario. The full ensemble of MEMs is included for both CMIP5 (7 models using IPSL forcings and 5 models using GFDL forcings, n = 12) and CMIP6 (9 models using IPSL forcings and 7 models using GFDL forcings, n = 16). Maps represent mean percentage change between 1990-1999 and 2090-2999 under a) CMIP5 and b) CMIP6; c) difference in percentage change between CMIPs; d) difference in percentage change between CMIPs for grid cells that showed the same direction of change; e) difference in percentage change between CMIPs for grid cells that changed from a decrease in CMIP5 to an increase in CMIP6 and f) vice versa. See Fig. S18 for the same comparison with the reduced set of MEMs.

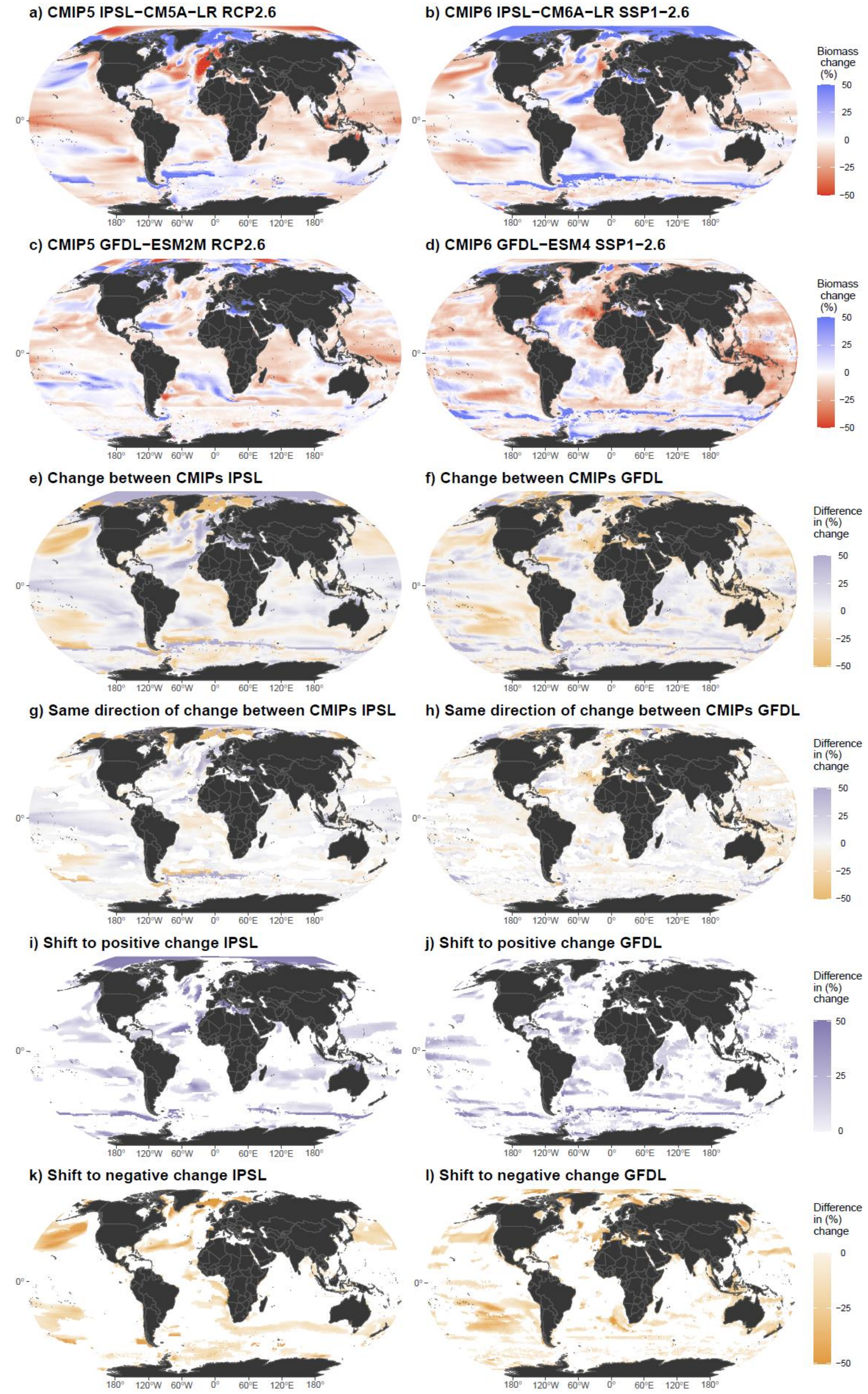
a) CMIP5 IPSL−CM5A−LR RCP2.6
b) CMIP6 IPSL−CM6A−LR SSP1−2.6
c) CMIP5 GFDL−ESM2M RCP2.6
d) CMIP6 GFDL−ESM4 SSP1−2.6
e) Change between CMIPs IPSL
f) Change between CMIPs GFDL
g) Same direction of change between CMIPs IPSL
h) Same direction of change between CMIPs GFDL
i) Shift to positive change IPSL
j) Shift to positive change GFDL
k) Shift to negative change IPSL
l) Shift to negative change GFDL
Biomass change (%)
Difference in (%) change
50
25
0
−25
−50
0°
180° 120°W 60°W 0° 60°E 120°E 180°

Supplementary Figure 6: Ensemble mean change in marine animal biomass across marine ecosystem models. Forcings from GFDL and IPSL under the strong-mitigation scenario. Maps represent mean percent change between 1990-1999 and 2090-2999 under a) CMIP5 IPSL; b) CMIP6 IPSL; c) CMIP5 GFDL; and d) CMIP6 GFDL. Remaining maps represent (e-f) difference in percent change between CMIPs; (g-h) difference in percentage change between CMIPs for grid cells that showed the same direction of change; (i-j) difference in percent change between CMIPs for grid cells that changed from a decrease in CMIP5 to an increase in CMIP6; vice versa (k-l).

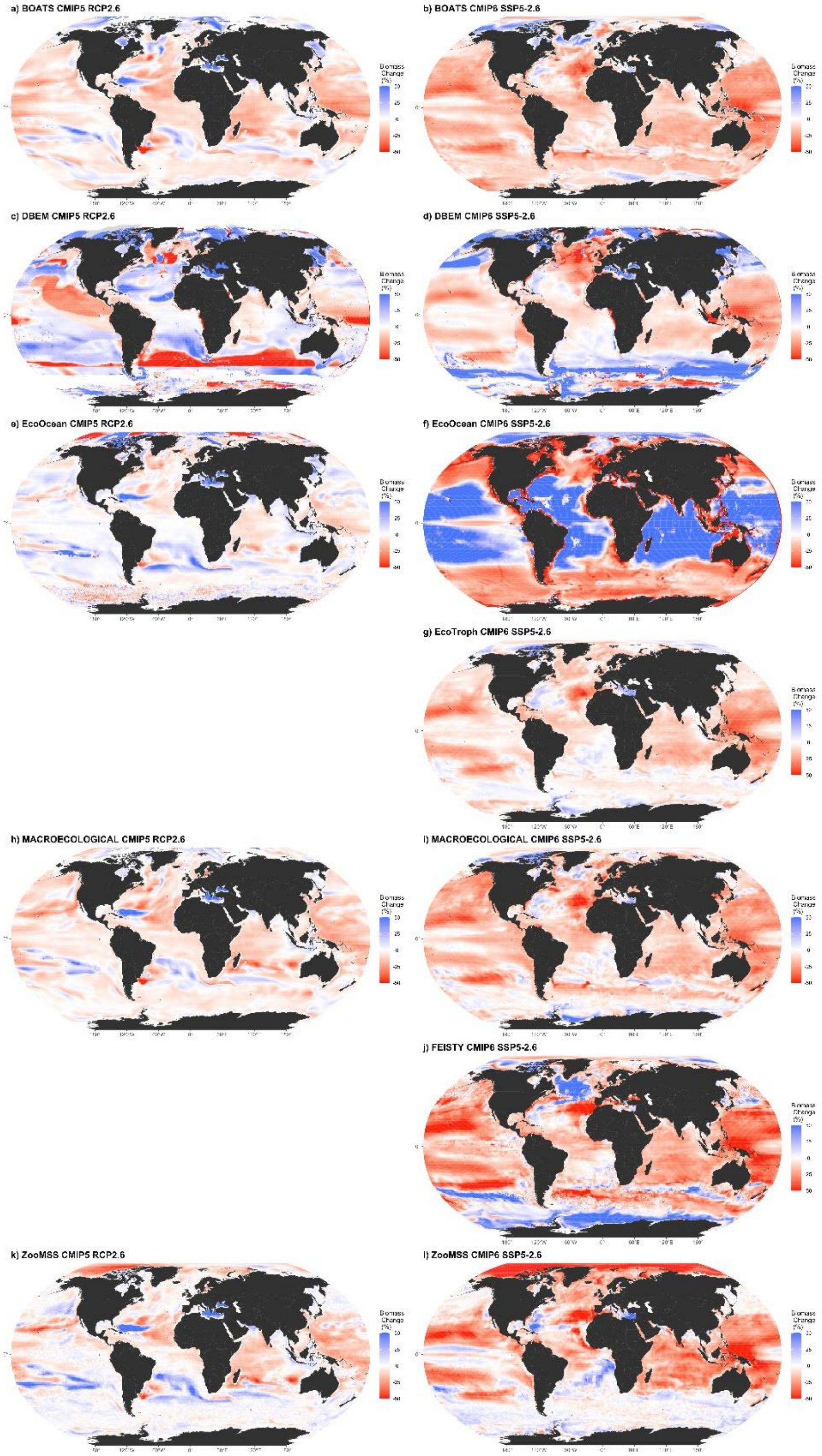
a) BOATS CMIP5 RCP2.6
b) BOATS CMIP6 SSP5-2.6
c) DBEM CMIP5 RCP2.6
d) DBEM CMIP6 SSP5-2.6
e) EcoOcean CMIP5 RCP2.6
f) EcoOcean CMIP6 SSP5-2.6
g) EcoTroph CMIP6 SSP5-2.6
h) MACROECOLOGICAL CMIP5 RCP2.6
i) MACROECOLOGICAL CMIP6 SSP5-2.6
j) FEISTY CMIP6 SSP5-2.6
k) ZooMSS CMIP5 RCP2.6
l) ZooMSS CMIP6 SSP5-2.6

Supplementary Figure 7: Change in marine animal biomass between 1990-1999 and 2090-2099 for marine ecosystem models under CMIP5 (left column) and CMIP6 (right column) forcings from GFDL under the mitigation scenarios.

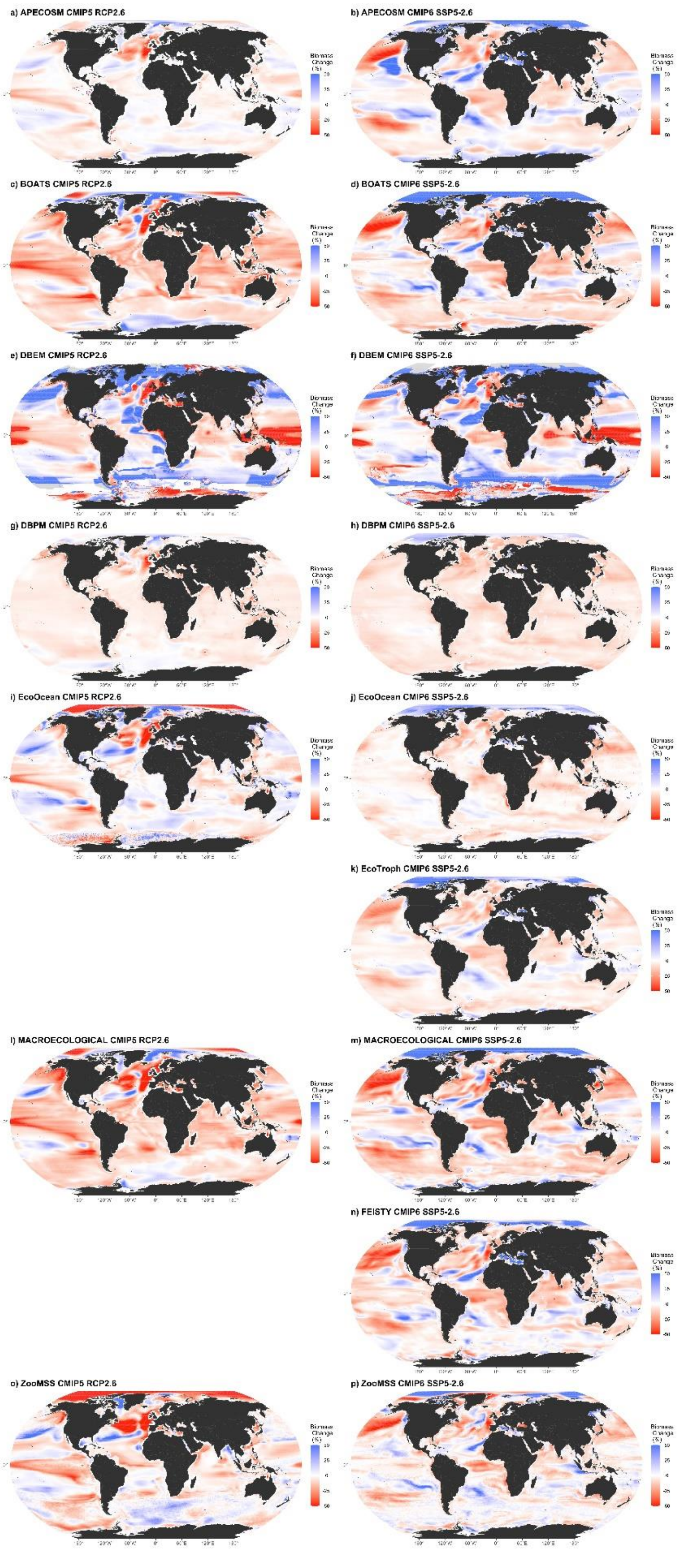
a) APECOSM CMIP5 RCP2.6
b) APECOSM CMIP6 SSP5-2.6
c) BOATS CMIP5 RCP2.6
d) BOATS CMIP6 SSP5-2.6
e) DBEM CMIP5 RCP2.6
f) DBEM CMIP6 SSP5-2.6
g) DBPM CMIP5 RCP2.6
h) DBPM CMIP6 SSP5-2.6
i) EcoOcean CMIP5 RCP2.6
j) EcoOcean CMIP6 SSP5-2.6
k) EcoTroph CMIP6 SSP5-2.6
l) MACROECOLOGICAL CMIP5 RCP2.6
m) MACROECOLOGICAL CMIP6 SSP5-2.6
n) FEISTY CMIP6 SSP5-2.6
o) ZooMSS CMIP5 RCP2.6
p) ZooMSS CMIP6 SSP5-2.6

Supplementary Figure 8: Change in total consumer biomass between 1990-1999 and 2090-2099 for marine ecosystem models under CMIP5 (left column) and CMIP6 (right column) forcings from IPSL under the mitigation scenarios.

c) Additional results from comparable set of MEM models under strong-mitigation scenario

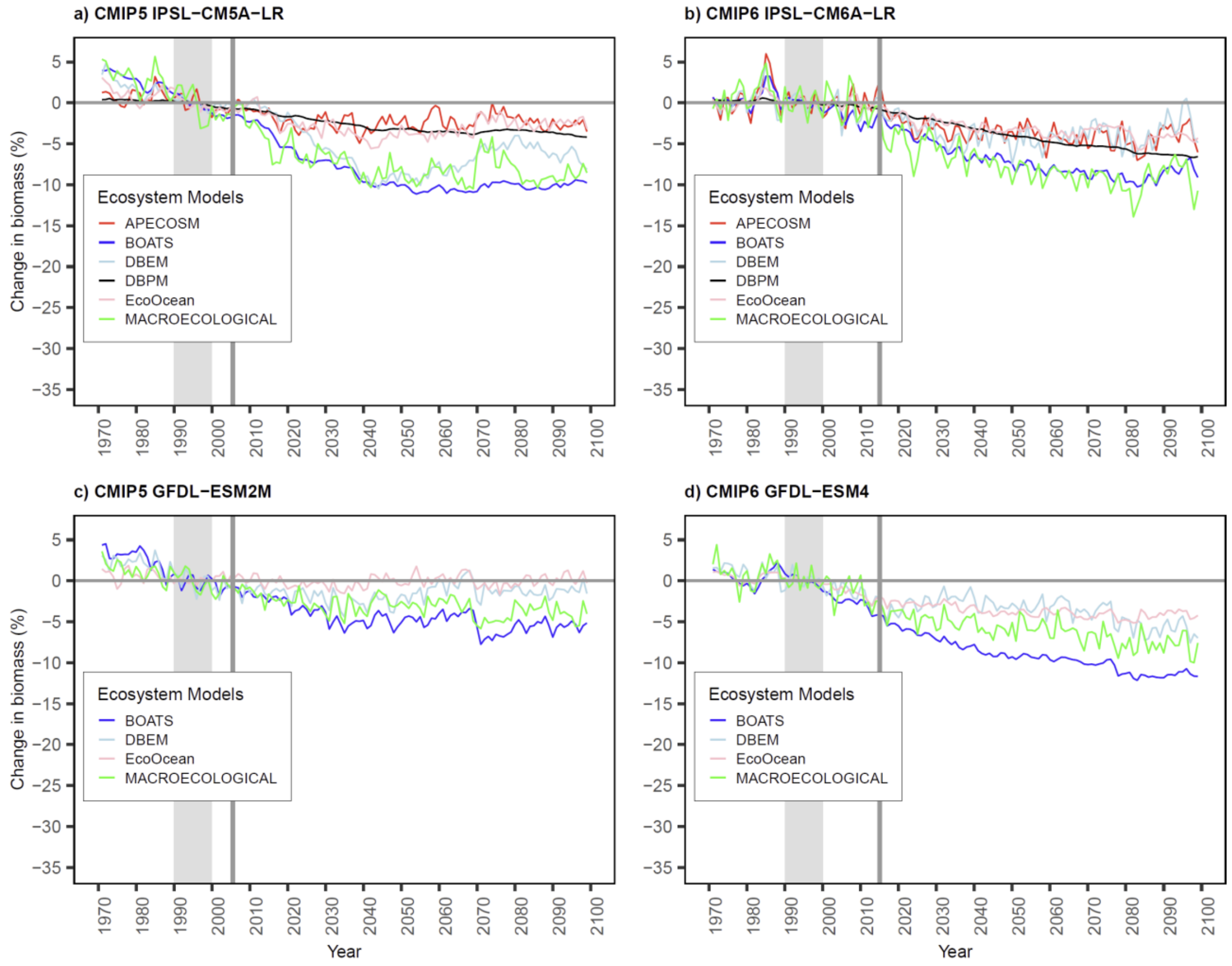

Supplementary Figure 9: Projected global change in total animal biomass across the comparable set of six marine ecosystem models (MEMs) that used both CMIP5 (a, c) and CMIP6 (b, d) forcings (n = 10; two MEMs only used IPSL). CMIP5 forcings from IPSL and GFDL under the strong-mitigation scenario RCP2.6 (a, c) and CMIP6 forcings from IPSL and GFDL under the strong-mitigation scenario SSP1-2.6 (b, d). All values are relative to the standardized reference period of 1990-1999. Vertical grey shaded area

indicates reference decade and vertical grey line indicates first year of projection (subsequent to historical period).

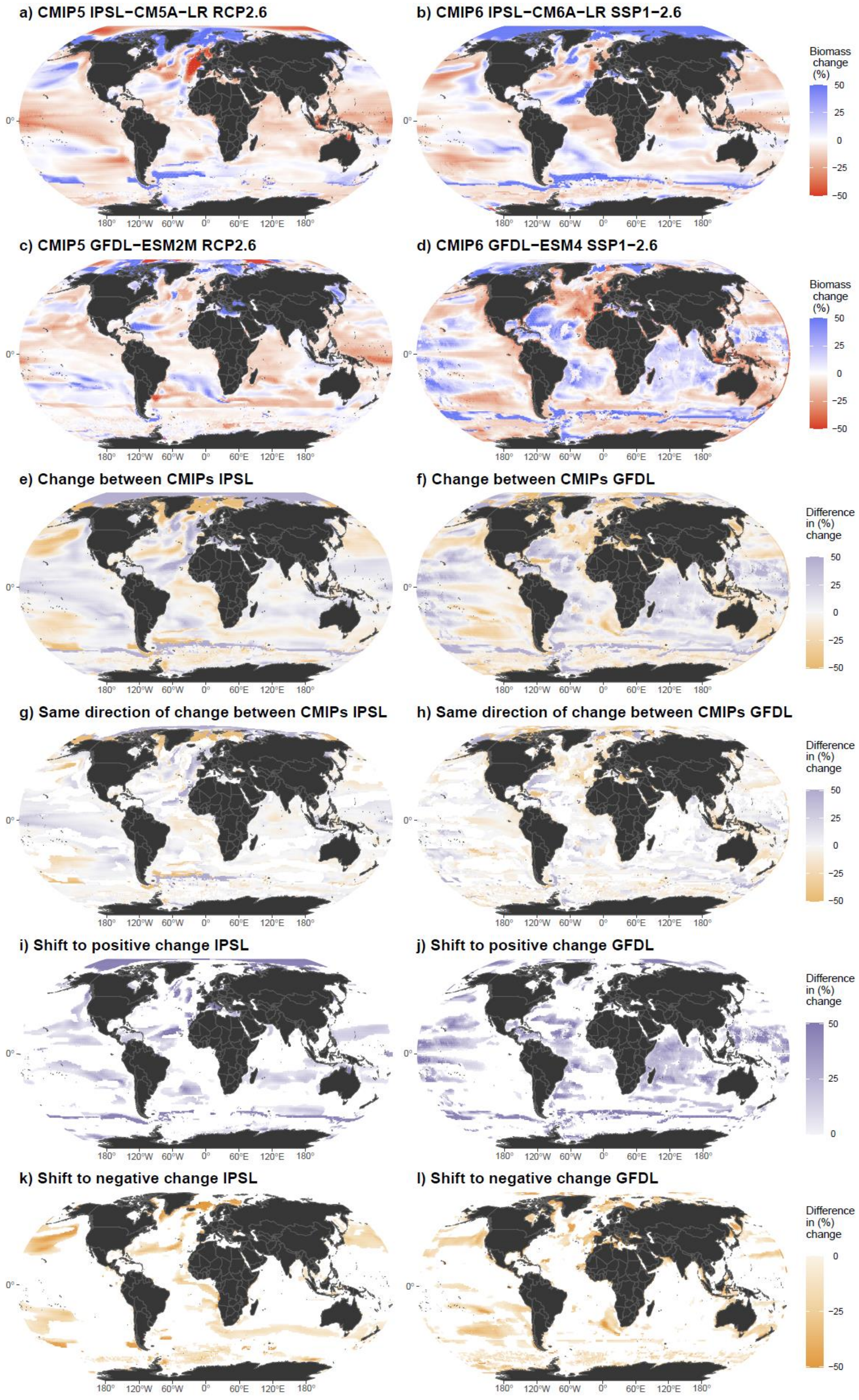
a) CMIP5 IPSL−CM5A−LR RCP2.6
b) CMIP6 IPSL−CM6A−LR SSP1−2.6
Biomass change (%)
50
25
0
−25
−50
c) CMIP5 GFDL−ESM2M RCP2.6
d) CMIP6 GFDL−ESM4 SSP1−2.6
Biomass change (%)
50
25
0
−25
−50
e) Change between CMIPs IPSL
f) Change between CMIPs GFDL
Difference in (%) change
50
25
0
−25
−50
g) Same direction of change between CMIPs IPSL
h) Same direction of change between CMIPs GFDL
Difference in (%) change
50
25
0
−25
−50
i) Shift to positive change IPSL
j) Shift to positive change GFDL
Difference in (%) change
50
25
0
k) Shift to negative change IPSL
l) Shift to negative change GFDL
Difference in (%) change
0
−25
−50
0°
180° 120°W 60°W 0° 60°E 120°E 180°

Supplementary Figure 10: Ensemble mean change in total animal biomass for the comparable set of marine ecosystem models that ran both CMIP5 and CMIP6 simulations. Forcings from GFDL and IPSL under the strong-mitigation scenario. Maps represent mean percent change between 1990-1999 and 2090-2999 under a) CMIP5 IPSL RCP2.6, b) CMIP6 IPSL SSP1-2.6, c) CMIP5 GFDL RCP2.6 and d) CMIP6 GFDL SSP1-2.6. Remaining maps represent (e-f) difference in percent change between CMIPs; (g-h) difference in percentage change between CMIPs for grid cells that showed the same direction of change; (i-j) difference in percent change between CMIPs for grid cells that changed from a decrease in CMIP5 to an increase in CMIP6; vice versa (k-l).

	Temperature	NPP / chlorophyll	Phytoplankton biomass	Zooplankton biomass	Export flux	Oxygen concentration	Current velocities	Photosynthetically active radiation	Salinity / water density	pH	Sea ice	Mixed-layer depth
APECOSM	x		x	x	x	x	x	x	x			
BOATS	x	x										
DBEM	x	x				x	x		x	x	x	x
DBPM	x		x									
EcoOcean	x		x									
EcoTroph	x	x		x								
FEISTY	x			x	x							
Macroecological	x	x										
ZooMSS	x	x										

Supplementary Table 1: A matrix of marine ecosystem models used in the analysis (left column) and forcing variables (top row). An *x* indicates that the ecosystem model uses Earth System Model output of that forcing variable; a blank space indicates that it does not.
