# Supplementary material for: Next-generation ensemble projections reveal higher climate risks for marine ecosystems
Source: Nat Clim Chang. 2021 Oct 21;11(11):973–81. doi: 10.1038/s41558-021-01173-9 (PMC8556156; doi:10.1038/s41558-021-01173-9)
Supplement: Supplementary file 2 — Reporting Summary [file 41558_2021_1173_MOESM2_ESM.pdf]

nature portfolio

Corresponding author(s): Derek P. Tittensor

Last updated by author(s): Aug 25, 2021

Reporting Summary

Nature Portfolio wishes to improve the reproducibility of the work that we publish. This form provides structure for consistency and transparency in reporting. For further information on Nature Portfolio policies, see our Editorial Policies and the Editorial Policy Checklist.

Statistics

For all statistical analyses, confirm that the following items are present in the figure legend, table legend, main text, or Methods section.

n/a	Confirmed	
☐	☒	The exact sample size (n) for each experimental group/condition, given as a discrete number and unit of measurement
☒	☐	A statement on whether measurements were taken from distinct samples or whether the same sample was measured repeatedly
☐	☒	The statistical test(s) used AND whether they are one- or two-sided<br>*Only common tests should be described solely by name; describe more complex techniques in the Methods section.*
☒	☐	A description of all covariates tested
☒	☐	A description of any assumptions or corrections, such as tests of normality and adjustment for multiple comparisons
☐	☒	A full description of the statistical parameters including central tendency (e.g. means) or other basic estimates (e.g. regression coefficient) AND variation (e.g. standard deviation) or associated estimates of uncertainty (e.g. confidence intervals)
☐	☒	For null hypothesis testing, the test statistic (e.g. F, t, r) with confidence intervals, effect sizes, degrees of freedom and P value noted<br>*Give P values as exact values whenever suitable.*
☒	☐	For Bayesian analysis, information on the choice of priors and Markov chain Monte Carlo settings
☒	☐	For hierarchical and complex designs, identification of the appropriate level for tests and full reporting of outcomes
☒	☐	Estimates of effect sizes (e.g. Cohen's d, Pearson's r), indicating how they were calculated

Our web collection on statistics for biologists contains articles on many of the points above.

Software and code

Policy information about availability of computer code

Data collection	Data were simulation outputs from individual marine ecosystem models, all cited in the main manuscript.
Data analysis	R version 4.0.3 was used to conduct the analysis of this data.

For manuscripts utilizing custom algorithms or software that are central to the research but not yet described in published literature, software must be made available to editors and reviewers. We strongly encourage code deposition in a community repository (e.g. GitHub). See the Nature Portfolio guidelines for submitting code & software for further information.

Data

Policy information about availability of data

All manuscripts must include a data availability statement. This statement should provide the following information, where applicable:

- Accession codes, unique identifiers, or web links for publicly available datasets
- A description of any restrictions on data availability
- For clinical datasets or third party data, please ensure that the statement adheres to our policy

All standardized forcing variables from the ESMs are available at doi: 10.48364/ISIMIP.575744.1; all outputs from the MEMs are available via ISIMIP (see: https://www.isimip.org/gettingstarted/data-access/).

Field-specific reporting

Please select the one below that is the best fit for your research. If you are not sure, read the appropriate sections before making your selection.

☐ Life sciences ☐ Behavioural & social sciences ☒ Ecological, evolutionary & environmental sciences

For a reference copy of the document with all sections, see nature.com/documents/nr-reporting-summary-flat.pdf

Ecological, evolutionary & environmental sciences study design

All studies must disclose on these points even when the disclosure is negative.

Study description	This study evaluates simulation outputs from marine ecosystem models forced by standardized earth-system models outputs from the CMIP5 and CMIP6 projects.
Research sample	The research sample is a set of marine ecosystem models from the FishMIP project (www.fishmip.org).
Sampling strategy	N/A
Data collection	ESM data were provided by ISIMIP; marine ecosystem model outputs were provided by individual modellers and coordinated by FishMIP.
Timing and spatial scale	Simulation outputs of the period 1970-2099 were analyzed. The spatial scale was global.
Data exclusions	No data were excluded.
Reproducibility	Models are reproducible in the sense that they can be re-run and outputs re-analyzed; all outputs are freely available.
Randomization	This is not relevant since we were not testing covariates.
Blinding	Blinding was not relevant to this study since we were not evaluating individual models versus one another but the whole set as an ensemble.

Did the study involve field work? ☐ Yes ☒ No

Reporting for specific materials, systems and methods

We require information from authors about some types of materials, experimental systems and methods used in many studies. Here, indicate whether each material, system or method listed is relevant to your study. If you are not sure if a list item applies to your research, read the appropriate section before selecting a response.

Materials & experimental systems

n/a	Involved in the study	
☒	☐	Antibodies
☒	☐	Eukaryotic cell lines
☒	☐	Palaeontology and archaeology
☒	☐	Animals and other organisms
☒	☐	Human research participants
☒	☐	Clinical data
☒	☐	Dual use research of concern

Methods

n/a	Involved in the study	
☒	☐	ChIP-seq
☒	☐	Flow cytometry
☒	☐	MRI-based neuroimaging
